# Discovery of Anion Insertion Electrochemistry in Layered Hydroxide Nanomaterials

**DOI:** 10.1038/s41598-019-39052-1

**Published:** 2019-02-21

**Authors:** Matthias J. Young, Tatyana Kiryutina, Nicholas M. Bedford, Taylor J. Woehl, Carlo U. Segre

**Affiliations:** 1 Applied Materials Division, Argonne National Laboratory, Argonne, Illinois 60439 USA; 2 Applied Chemicals and Materials Division, National Institute of Standards and Technology, Boulder, Colorado 80305 USA; 3 Department of Biomedical, Biological, and Chemical Engineering, University of Missouri, Columbia, MO 65211 USA; 4 Department of Chemistry, University of Missouri, Columbia, MO 65211 USA; 5 School of Chemical Engineering, University of New South Wales, Sydney, NSW 2052 Australia; 6 Department of Chemical and Biomolecular Engineering, University of Maryland, College Park, Maryland 20742 USA; 7 Department of Physics, Illinois Institute of Technology, Chicago, Illinois 60616 USA

## Abstract

Electrode materials which undergo anion insertion are a void in the materials innovation landscape and a missing link to energy efficient electrochemical desalination. In recent years layered hydroxides (LHs) have been studied for a range of electrochemical applications, but to date have not been considered as electrode materials for anion insertion electrochemistry. Here, we show reversible anion insertion in a LH for the first time using Co and Co-V layer hydroxides. By pairing *in situ* synchrotron and quartz crystal microbalance measurements with a computational unified electrochemical band-diagram description, we reveal a previously undescribed anion-insertion mechanism occurring in Co and Co-V LHs. This proof of concept study demonstrates reversible electrochemical anion insertion in LHs without significant material optimization. These results coupled with our foundational understanding of anion insertion electrochemistry establishes LHs as a materials platform for anion insertion electrochemistry with the potential for future application to electrochemical desalination.

## Introduction

A new desalination method which is scalable, low cost, and leverages renewable energy has been identified as the #1 most critical advance needed for sustainable global development^1^. Capacitive deionization (CDI) is an emerging electrochemical desalination technology which shows promise to fulfill this need, but currently out-performs reverse osmosis only for low salinity feed waters (<3 g/L)^2^, limited largely by the performance of existing electrode materials. Recent advances have improved upon conventional carbon electrodes commonly used in these devices by employing faradaic reactions and pairing a cation insertion electrode with an anion insertion electrode to enhance CDI capacity and efficiency^3,4^. High rate cation insertion materials derived from battery and supercapacitor technology which are stable in water are well suited for this application, but few materials are known which undergo electrochemical *anion* insertion. Due to charge balance constraints during deionization, a cation removal electrode should be paired with an anion removal electrode in a conventional cell construction. Recent work has overcome this restriction using two cation insertion electrodes separated by an anion exchange membrane (AEM) in a different mode of operation^5–7^. Although AEM development is an active area of research with recent advances^8^, the performance limitations of AEMs are a significant bottleneck to this approach. Identifying new materials for anion insertion electrochemistry is a necessary first step to enable enhanced desalination efficiency using membrane-free CDI cell designs. Moreover, the study of anion insertion electrode materials is a new frontier which promises to lead to other exciting electrochemical technologies including anion-based batteries or enhanced anion exchange membranes.

To date, silver is the leading material which has been used to enhance anion uptake during CDI. Silver reacts according to Ag + Cl^−^ ↔ AgCl + e^−^ and is used as an electrochemical reference electrode^9^, but has a number of limitations for practical use in CDI, including high cost, limited kinetics for sub-surface conversion^10^, oxidation side-reaction to form silver oxide^11^, and toxicity of nanoparticulate Ag and dissolved Ag^+^ ^12^. While AgCl has a comparatively low aqueous solubility of 0.19 mg AgCl/100 g H_2_O, the high solubility of other metal chlorides in water bar the use of metal/metal chloride reactions for aqueous anion electrochemistry in general^13^. Redox polymers based on, for example, poly-p-phenylene^14,15^ or quaternary amines such as viologen^16^ show promise as anion insertion electrode materials, but have not been studied extensively. Some recent studies have also identified anion insertion in LaMnO_3_^17^ and BiOCl^18^ under applied potential. However, compared with the variety of cation insertion materials^19–22^, relatively few materials have been identified which incorporate anions under applied potential. Identifying new anion insertion materials will help to fill a void in the materials innovation landscape, particularly in emerging areas of technological importance such as CDI^1^.

We contend that layered hydroxides (LHs) may be a promising option for anion insertion electrochemistry considering their use as anion exchange materials^23^, as well as their facile synthesis and modularity of possible chemical composition^24^ which allow for tunable (electro) chemical properties. The LH atomic structure consists of alternating layers of (a) positively charged planes of metal centers which are octahedrally coordinated to hydroxyl groups and (b) interplanar anions and water. The metal hydroxide planes in LHs can be comprised of a single metal center (e.g. cobalt hydroxide with root formula Co(OH)_2_, which exists in both α and β phases^25^), two metal centers, termed layered double hydroxides (LDHs), (e.g. naturally occurring hydrotalcyte which contains positively charged layers of root formula Mg_3_Al(OH)_8_^+^), or more than two metal centers (layered poly-hydroxides). Metal centers in the metal hydroxide planes are a blend of divalent (e.g. Mg, Co) and trivalent (e.g. Al, Cr, V, Co) cations. Interplanar anions in the LH structure balance charge with tri-valent metal cations in the metal hydroxide planes and cohere the planes together into nanoplatelet stacks. In this work we explore whether these interplanar anions are able to reversibly intercalate in the LH structure during electrochemical cycling. In addition to anion exchange applications mentioned above, LH nanomaterials have been studied for drug delivery^26^, and have garnered recent interest for their remarkable enhancements to a wide range of electrochemical applications^27^ including water electrolysis^28,29^, photoelectrocatalysis^30,31^, supercapacitors^32–35^, and batteries^36,37^. A limited number of studies have also evaluated calcined LHs in CDI electrodes and observed salt removal^38,39^. However, the electrochemical mechanisms occurring in LHs are not well understood, and the role of anions in their electrochemical performance has not been established.

We employ advanced *in situ* characterization during electrochemical operation and *ab initio* material modeling to probe anion insertion electrochemistry in LH nanoplatelets. The unified electrochemical band-diagram (UEB) framework^40,41^ enables modeling of the thermodynamics of anion insertion and associated structural changes under applied bias despite the lack of a convenient computational anion reference. Furthermore, coupling computational results within the UEB framework with *in situ* experimental measurements including quartz crystal microbalance (QCM), high energy X-ray diffraction (HE-XRD) coupled to atomic pair distribution function (PDF) analysis and X-ray absorption spectroscopy (XAS) provides compelling evidence of anion insertion electrochemistry in LHs for potential future use in water desalination applications.

## Results and Discussion

### Identifying LH Compositions with Desired Conduction Band Edge Energies

In this work, we examine cobalt-containing LHs for anion insertion. The seminal work identifying the LDH structure and synthesis^42–44^ and demonstrating particle size control^45^ are based on Mg-Al compositions. While the Mg-Al LDH synthesis is robust and well-studied, we do not expect Mg-Al LDHs to be useful as anion insertion electrodes because Mg and Al do not undergo redox electrochemistry within the potential limits of water stability. Incorporating metal centers into the LH structure which are redox-active within the potential limits for water stability provides a strategy for enabling aqueous anion-insertion electrochemistry. Here, we focus on Co-containing LHs because successful Co-LDH syntheses are known^24,46,47^, and Co-containing oxides, (*e*.*g*. LiCoO_2_) have been shown to undergo redox reactions within the potential stability limits of aqueous electrolytes^48^.

**Figure 1:**
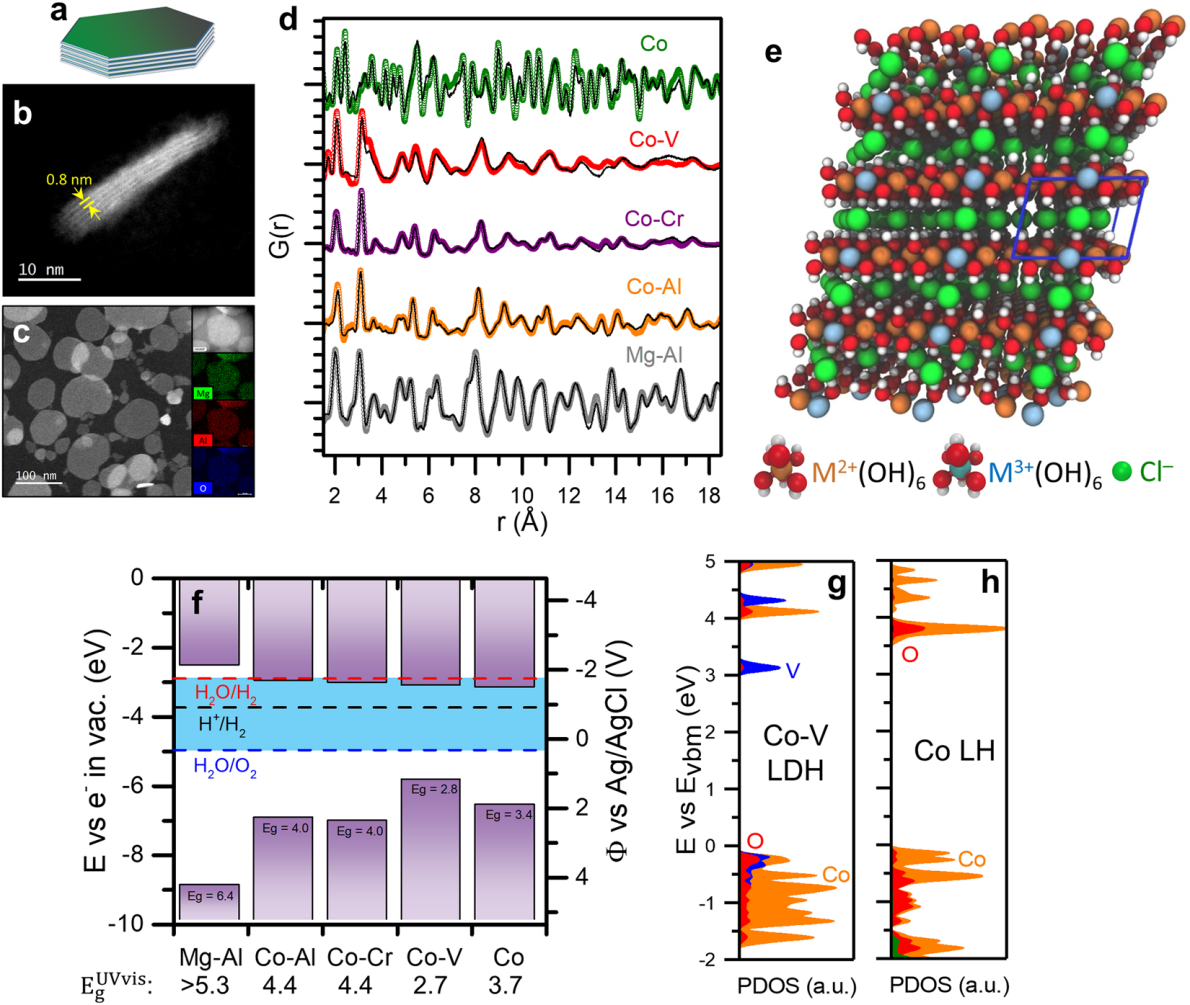
Tuning conduction band edge of layered hydroxides. (**a**) Cartoon schematic of isolated LH nanoparticle based on (**b**) high resolution ADF-STEM image of Co-Al LDH showing ~5 nm nanoplatelet thickness with visible LH planes and (**c**) ADF-STEM micrograph of Mg-Al LDH showing nanoplatelet morphology with EDS mapping of Mg, Al, and O. (**d**) Pair distribution function (PDF) analysis of various LH compositions with reverse Monte Carlo (RMC) fitting showing equivalent structure among various LH compositions. (**e**) Visualization of relaxed Mg-Al LH structure from *ab initio* modeling with 2:1 M^2+^:M^3+^ ratio, containing metal hydroxide planes and interplanar anions. (**f**) Band edge locations of various LH structures from *ab initio* modeling within an electrochemical reference frame, compared with experimental band gap values from UV-Vis spectroscopy, as well as the projected density of states (PDOS) as a function of the energy above the valence band maximum energy (E vs E_vbm_) for (**g**) Co-V and (**h**) Co LH from *ab initio* modeling.

Using coprecipitation of metal chloride salts in strong basic solution^44^, we synthesize aqueous suspensions of LH nanoparticles (ESI Section A). LHs spontaneously form a nanoplatelet morphology as depicted in Fig. 1a–c. Upon dropcasting, these nanoparticles coalesce into nearly uniform coatings on various flat electrode surfaces^49^. A nanoplatelet morphology was observed for all of the LDH compositions examined in this work. In most cases, nanoplatelets were ~100 nm in diameter and 2–5 nm thick, as demonstrated in annular dark field (ADF) scanning transmission electron microscopy (STEM) micrographs for Co-Al and Mg-Al compositions in Fig. 1b,c, respectively. The Mg-Al STEM micrograph also contains energy dispersive X-ray spectroscopy (EDS) mapping showing uniform blending of Mg and Al. STEM micrographs of the Co LH (Fig. S1) and Co-V (Fig. S2) with EDS analysis (Fig. S3) are provided in ESI Section B. The metal hydroxide layers and particle thickness were observed to be consistent across various LDH compositions, and are particularly clear for the Co-Al LH in Fig. 1b, which depicts a ~5 nm thick particle with a layer separation of ~8 Å.

Using atomic pair distribution function (PDF) analysis of high energy X-ray diffraction (HE-XRD) patterns as depicted in Fig. 1d, we observe an equivalent atomic structure among the Mg-Al, Co-Al, Co-Cr, and Co-V LDHs studied in this work. PDF analysis was performed for Mg-Al (Fig. S4) and Co-LHs (Fig. S5). The PDF calculation steps, including conventional XRD diffractograms with reference peaks are reported in ESI Section C. PDF analysis is useful here due to the nanoscale of the LH platelets and the fine structural detail of interest during *in situ* measurement of anion insertion (see below). Atomic PDFs provide sub-Ångstrom structural details in terms of real-space atomic distances^50,51^, and more intuitively showcase the structural features present in the LDH compositions (Mg-Al, Co-Al, Co-Cr, and Co-V). Main atomic pair features at ~2, ~3, and ~8 Å correspond to first-coordination sphere metal-oxygen, metal-metal, and interlayer metal-metal pair distances, respectively, for all LDHs examined. Reverse Monte Carlo (RMC) fits^52^ (thin black lines in Fig. 1d) were performed using starting LH structures determined from *ab initio* calculations, an example of which is depicted in Fig. 1e. An example RMC-fitted structure is shown in Fig. S4 for the Mg-Al LDH. RMC-fitted PDFs are in close agreement with measured PDFs indicating formation of the LDH structure in all cases. The interlayer metal-metal peak at ~8 Å for the Co-Al LDH in Fig. 1d is also consistent with the interplanar spacing observed by STEM in Fig. 1b. Synthesis of a LH using only Co(II) without M^3+^ cations resulted in a qualitatively different PDF (top of Fig. 1d), which was fit using a Co(OH)_2_ LH structure without interplanar anions. See the ESI section C and D for computational details regarding RMC fitting and *ab initio* modeling, respectively.

Initially, we hypothesized that the conduction band edge energy of the LH would determine the potential at which anion insertion occurs in an LH. As a negative bias is applied and increases the fermi energy of the electrode, we expected that electrons would transfer into the LH when the conduction band edge energy is reached, driving reduction of the LH and leading to anion expulsion according to

1 $${\rm{M}}({\rm{III}}){({\rm{OH}})}_{2}{\rm{Cl}}+{{\rm{e}}}^{-}\rightleftharpoons {\rm{M}}({\rm{II}}){({\rm{OH}})}_{2}+{{\rm{Cl}}}^{-}$$

which could again be driven toward the reactants (anion insertion) under reverse polarization. The theoretical charge storage capacity consistent with this mechanism is dependent on the specific composition and is 88 mAh/g for the Co_2_V(OH)_6_Cl LDH. Based on this mechanistic picture, we computationally evaluated the band edge positions for the LHs synthesized in Fig. 1d (rigid band model) using an idealized 2:1 M^2+^-M^3+^ molar ratio LDH structure depicted in Fig. 1e. By calculating the Fermi level energy of the bulk material with respect to an electron in vacuum in this way (Fig. S6), we determine the absolute band edge positions as presented in Fig. 1f. Our computational results show that composition strongly impacts the electronic properties of the LH structures, which are predicted to have band gaps ranging from 2.8 eV (Co-V) to 6.4 eV (Mg-Al). We also note that even though the composition of experimental LH structures does not match the ideal 2:1 structure, the computed band gaps for the LH structures in Fig. 1f agree (within 0.4 eV) with experimental band gaps reported beneath Fig. 1f. These band gaps were determined using ultraviolet-visible (UV-Vis) spectroscopy and Tauc plot analysis (Fig. S7) as described in ESI section E. Of the compositions presented in Fig. 1d, we selected the Co-V and Co LHs for further analysis because our *ab initio* modeling predicted their conduction band edge positions lie at the lowest energies. Projected density of states (PDOS) for Co-V and Co LHs are presented in Fig. 1g,h, respectively. The conduction band edge of the Co-V LDH is predicted to be V-character, whereas the conduction band edge of the Co LH is predicted to be Co-character. To a first approximation, we expected these LH constituent species to take on electron density at the conduction band edge energies under applied negative bias and drive anion expulsion according to reaction 1.

### EQCM Observation of Electrochemical Anion Insertion

**Figure 2:**
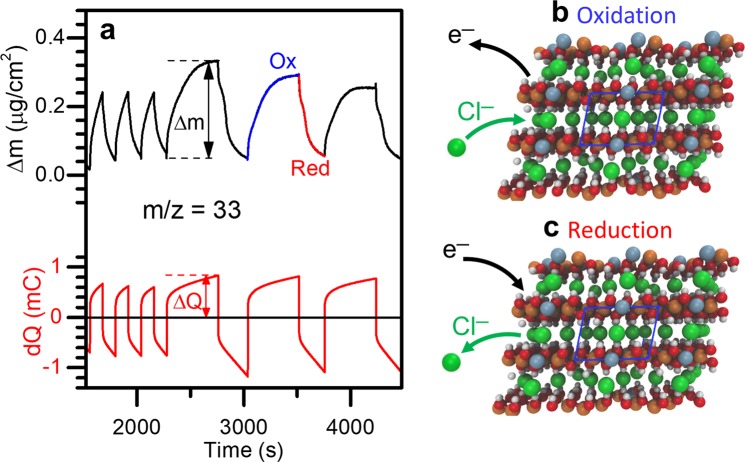
Cl^−^ insertion in Co LH observed by EQCM. (**a**) EQCM measurement on Co LH cycled under potentiostatic control between −0.4 V vs. Ag/AgCl and +0.2 V vs. Ag/AgCl, indicating (**b**) anion insertion under oxidizing potential and (**c**) anion removal under reducing potential.

Electrochemical evaluation of the Co-V and Co LHs yielded a surprising result. While we do observe reversible anion insertion/expulsion electrochemistry in both the Co-V and Co LHs, it initiates as anion insertion under oxidizing (*positive)* potentials rather than anion expulsion under reducing (*negative)* potentials as we hypothesized. Presented in Fig. 2a is electrochemical quartz crystal microbalance (EQCM) trace for the Co-only LH. In these EQCM measurements the working electrode is the titanium surface of a quartz resonator, which can be used to measure *in situ* mass changes and charge transfer simultaneously during electrochemical operation. Here we observe compelling evidence of anion insertion when cycling under positive bias of +0.2 V vs. Ag/AgCl and anion expulsion at −0.4 V vs. Ag/AgCl. For the first three cycles in Fig. 2, each potential was held for a duration of 120 s, while for the successive three cycles, the potential was held at +0.2 V vs. Ag/AgCl for 480 s, and −0.4 V vs. Ag/AgCl for 240 s. For all six cycles, we observe a monotonic mass *gain* under positive potential (oxidation) and mass *loss* under negative potential (reduction). This is qualitatively consistent with anion insertion: under applied positive bias, Cl^−^ is drawn into the Co LH structure as depicted in Fig. 2b, and under applied negative bias Cl^−^ is expelled from the Co LH structure as depicted in Fig. 2c.

The mass of Co LH used in Fig. 2a was 57.8 µg, as calculated from the decrease in resonant frequency of the EQCM crystal after dropcasting and drying the Co LH nanoparticles assuming Sauerbrey behavior for the dropcast Co LH film. During the first three cycles in Fig. 2, the Co LH was charged to an average of 0.76 mC during reduction and 0.64 mC during oxidation, which correspond to 3.65 mAh/g and 3.09 mAh/g, respectively. During the successive three cycles, the Co LH was charged to an average of 1.12 mC on reduction s and 0.79 mC on oxidation, which correspond to 5.38 mAh/g and 3.8 mAh/g, respectively. The region marked with dashed lines and arrows in Fig. 2 yields a calculated m/z of 32.9 g/mol e^−^, in close agreement with the value of 34.5 g/mol e^−^ expected for stoichiometric Cl^−^ insertion. The six cycles shown in Fig. 2 yield an average m/z of 30.2 ± 2.8 g/mol e^−^ on the oxidizing sweep, and 23.0 ± 2.6 g/mol e^−^ on the reducing sweep. The lower value on the reducing sweep indicates some irreversibility, which we attribute to delamination of LH sheets as anions are driven out as well as ion-decoupled electron transfer as described elsewhere^53^. This irreversibility is also reflected in the low coulombic efficiency of 85% over the first three cycles, and 70% over the successive three cycles in Fig. 2a.

Despite some irreversibility, this data provides strong support for Cl^−^ insertion in Co LH under oxidizing (positive) potentials. The theoretical charge storage capacity for anion insertion in the Co LH according to

2 $$Co{(OH)}_{2}+C{l}^{-}\rightleftarrows Co{(OH)}_{2}Cl+{e}^{-}$$

is 209 mAh/g. The Co LH was charged to only a small fraction of the theoretical specific capacity. For the first three cycles shown in Fig. 2 the Co LH was charged to ~3 mAh/g, and for the final three cycles in Fig. 2 the Co LH was charged to ~5 mAh/g. We note this is a largely unoptimized material which was dropcast onto EQCM crystals with no binder or conductive additive to isolate and study the electrochemical behavior of the LH nanoplatelets. Future efforts to measure the desalination performance of this material will benefit from ongoing materials design work and electrode formulation improvements.

### EQCM Study of LHs Under Reducing Potentials

**Figure 3:**
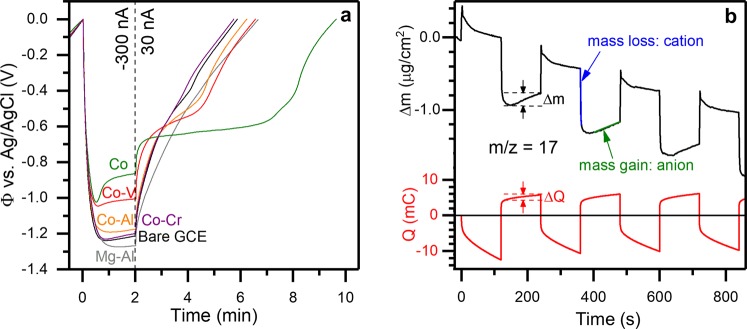
Reduction of layered hydroxides. (**a**) Experimental electrochemical measurement of potential (Φ) versus time under galvanostatic operation, indicating reductive electrochemistry for Co, Co-Al, and Co-V LHs. (**b**) EQCM measurement of mass changes (Δm) and charge transfer (Q) during potentiostatic cycling of Co-V LDH between −1.0 V and −0.5 V vs. Ag/AgCl, identifies rapid cation-based mechanism followed by slower anion-based mechanism under applied negative bias.

To contrast the anion insertion mechanism observed under positive potential conditions in Fig. 2, here we examined the electrochemical behavior of various LHs under negative potential conditions. Each LH was dropcast onto a glassy carbon electrode (See ESI Section F) and evaluated using cyclic voltammetry (Fig. S8), followed by galvanostatic measurement (Fig. 3a). Under applied negative current, the Fermi level in each LH (Fig. 1d,e) increases, corresponding to more negative potentials for the experimental measurements in Fig. 3a. On the reduction steps in Fig. 3a, a current of −300 nA is held for 2 min, corresponding to 36 µC. By the rigid band description in Fig. 1f, electrons are expected to transfer into unoccupied V orbitals (Fig. 1g) in the Co-V structure at a potential of −1.5 V vs Ag/AgCl (Fig. 1f). While we do observe a plateau in potential for Co-V LH in Fig. 3a, it is at a significantly lower potential of ~−1.0 V vs. Ag/AgCl, at odds with expected conduction band positions based on the rigid band description in Fig. 1f. Additionally, we observe a plateau at a similar potential to the Co-V LDH for the Co-Al LDH and a plateau at a dramatically lower potential for the Co-only LH, neither of which are consistent with the conduction band edge positions based on the rigid band description in Fig. 1f. We attribute the irreversible charge transfer at −1.2 V vs. Ag/AgCl for the bare glassy carbon electrode (GCE), as well as Mg-Al LDH, and Co-Cr LDH measurements to the onset of the hydrogen evolution reaction^9^, which precludes the study of these LHs at more reducing potentials. Upon switching to a positive current at a time t = 2 min in Fig. 3a, the Fermi level decreases, and electrons transfer back out of the Co-V, Co-Al, and Co LH structures, leading to plateaus in potential under positive current as depicted in Fig. 3a. On these oxidation steps, a current of 30 nA was held until the potential reached 0 V vs. Ag/AgCl. The width of the plateau corresponds to the amount of charge transferred. We observe the widest plateau for the Co LH, corresponding to 13 µC (36% coulombic efficiency), while narrower plateaus, corresponding to 7–8 µC (~20% coulombic efficiency) are observed for the Co-V, and Co-Al LHs.

We again employ EQCM here to understand the electrochemical behavior of these LHs under negative bias. The results from an EQCM measurement on the Co-V LH platelets is depicted in Fig. 3b. The mass of Co-V LDH used in Fig. 3b was 229.5 µg, as calculated from the decrease in resonant frequency of the EQCM crystal after dropcasting and drying the Co-V LDH nanoparticles assuming Sauerbrey behavior for the dropcast film. The larger ΔQ values we observe in Fig. 3b versus Fig. 2a arise in part from the larger mass of dropcast LH nanoparticles in Fig. 3b versus Fig. 2a, and in part because of different electrochemical behavior within the different potential windows. We attribute the overall decrease in mass for the Co-V LDH over the 800 s interval shown in Fig. 3b to the delamination of the LH particles and dissolution of V (*vide infra*) during operation. We note that electrode stability is of critical importance for long-term water desalination, which is currently being addressed and will be the subject of future publications. For instance, delamination could be mitigated using a laminate electrode with a polymeric binder to immobilize the LH particles. We omitted binder in this work to isolate the electrochemical behavior of the LHs. Future work will explore cycling and desalination performance of these materials using laminate electrodes but is outside the scope of this work.

For anion insertion charge storage, one would expect a mass loss under applied negative bias and a mass gain under applied positive bias. The predominant signal we measure in Fig. 3b is the inverse of this, with a mass *gain* under applied negative bias (−1.0 V vs. Ag/AgCl), and a mass *loss* under applied positive bias (−0.5 V vs. Ag/AgCl). This is at odds with anion insertion, and suggests a cation-based mechanism, such as the reversible formation of Co or V vacancies or the insertion of electrolyte cations (*e*.*g*. Na^+^) into the structure. The m/z over this cation-based region was calculated to be 21 g/mol e^−^ which is in close agreement with sodium insertion (23 g/mol e^−^) or formation of trivalent cobalt vacancies (20 g/mol e^−^). However, this cation-mediated process occurs contemporaneously with a slower process following the initial mass spikes, highlighted with the dashed lines in Fig. 3b, which is consistent with an anion-based mechanism. The unknown contributions of these two processes during the initial few seconds of oxidation prevent conclusive analysis of the cation-based mechanism. However, the steady-state mass-to-charge ratio (m/z) we derive from the slopes highlighted in Fig. 3b is m/z = 17 g/mol e^−^, which agrees precisely with the mass-to-charge ratio expected for OH^−^ and strongly suggests that OH^−^, not Cl^−^, is the species involved in this slower anion-based process.

### UEB Modeling to Refine Mechanistic Picture of LH Electrochemistry

To better understand this electrochemical behavior, we expand upon the rigid band description in Fig. 1f and employ the UEB construct^40,41,54^ to model the thermodynamics of charged defects in LH structures under applied bias. (See refs^40,41,54^. and ESI Section D for further information.) We emphasize that conventional ΔE_rxn_ calculations versus a metallic reference commonly employed for studying cation insertion^55^ are not well-suited to study this system because (a) no convenient electrochemical reference (*e*.*g*. bulk metal) is available for Cl^−^ anions, and (b) some of the relevant reactions involve multiple atomic species. The UEB construct provides a means of overcoming these obstacles. Within the UEB construct, an applied bias shifts the Fermi level within the band gap of these LH structures and drives structural perturbations and the formation of charged defects (including multi-species defects) and identifies redox potentials versus an electrochemical reference based on the energy levels of electronic states inside the band gap. Specifically, in UEB defect plots, the formation energy of a given defect, ΔE_f_, is calculated for various defect charge states while accounting for the applied bias and electrolyte pH as described in the Materials and Methods (ESI Section D). A structural change is predicted to exist when a given defect has a ΔE_f_ < 0, and that defect is expected to give rise to charge transfer when it changes charge state—corresponding to the kinks marked with symbols. Using the UEB computational framework we are able to predict and understand the electrochemical behavior we observe experimentally, as discussed below.

**Figure 4:**
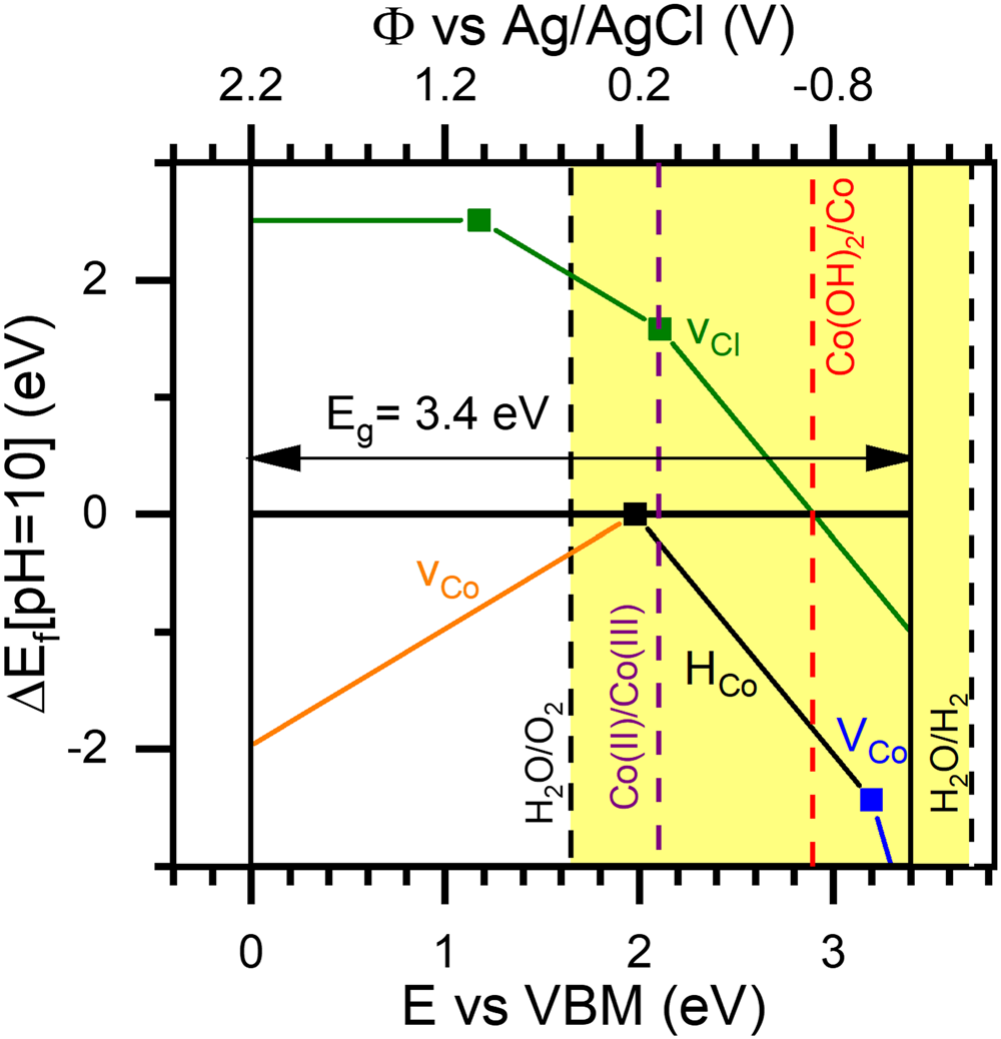
UEB model of Co_3_(OH)_6_Cl. *Ab initio* modeling of formation energy (ΔE_f_) at a pH of 10 versus energy above the valence band maximum (E vs VBM) as well as potential (Φ) versus Ag/AgCl for Co_3_(OH)_6_Cl identifies cobalt vacancy (*v*_*Co*_), and chloride vacancy (*v*_*Cl*_) mediated electrochemical mechanisms. Vertical dashed lines represent H_2_O (black) stability and Co (purple and red) phase change reactions based on a Pourbaix construct.

STEM with EDS mapping and ICP-OES analysis (ESI Section A and G, respectively) of the Co-V sample indicate a blend of V-rich Co_3_V(OH)_8_Cl (Co_3_V LDH) and V-deficient Co_3_(OH)_6_Cl. In order to understand the electrochemical behavior of the Co-V LDH in Fig. 3b, we study both V-rich Co_3_V LDH and V-deficient Co_3_(OH)_6_Cl LH within the UEB construct. We note that neither of these structures alone perfectly captures the experimental Co-V LH structure, rather these compositions bound the range of chemical composition observed in the Co-V LH. Combining the modelling results from both reference points allows us to understand the range electrochemistry occurring in the Co-V sample. We examine vacancy, substitution, and interstitial point defects at various charged states within these structures. In ESI Section H we conclude that V-rich LDH domains in the Co-V LH sample are not expected to contribute to the reversible charge storage we observe in Fig. 3a,b — in Fig. S9, no defects in the Co_3_V LDH are predicted to be thermodynamically favorable and change charge state in the potential range of {0, −1.0} V vs. Ag/AgCl. Based on these calculations, electrochemical insertion of Cl^−^ into the Co_3_V LDH requires a potential >0.4 V vs. Ag/AgCl, and anion expulsion requires a potential <−1.6 V vs. Ag/AgCl. Additionally, V vacancies (*v*_*V*_) are predicted to be highly favorable (ΔE_f_ < −2 eV), driving the conversion of Co_3_V LDH to Co LH. Considering this, the anion insertion behavior in the Co-V LH sample likely arises from Co LH domains. We examine the UEB construct of Co_3_(OH)_6_Cl as presented in Fig. 4. Cobalt hydroxide is known to form as either a α-Co(OH)_2_ phase with interlayer Cl, or a Cl-free β-Co(OH)_2_ phase depending on synthesis conditions^25^. As-synthesized, the atomic pair distances measured in the Co LH are consistent with β-Co(OH)_2_ (top of Fig. 1d as discussed above). However, the V-deficient regions in the Co-V LDH are expected to more closely match α-Co(OH)_2_ based on the interlayer spacing for the Co-V sample in Fig. 1d. Furthermore, the Co LH is expected to contain interlayer Cl^−^ upon anion insertion under positive bias. Here, we employ Co_3_(OH)_6_Cl as a model system to enable convenient modeling of chloride insertion and expulsion in cobalt hydroxide. We note that the structure we use for this modeling nominally contains a mixture of both Co(II) and Co(III) and describes a partially oxidized Co(OH)_2_ structure with 1/3 Co(III) centers and stoichiometric balance of Cl^−^.

The predicted electrochemistry of the Co_3_(OH)_6_Cl using the UEB construct is in line with our experimental observations for both the Co-V and Co LHs. First, UEB calculations on Co_3_(OH)_6_Cl provide an explanation for the cation-mediated mechanism we observe for the Co-V sample in Fig. 3c. Under reducing current, at −1 V vs. Ag/AgCl, protons on Co vacancies (*H*_*Co*_) are predicted to exchange for V on Co vacancies (*V*_*Co*_), giving rise to charge transfer and cation uptake. In other words, V ions which have dissolved from the Co-V sample will reinsert into Co vacancies under applied negative bias. When the current is reversed to oxidizing conditions, the V is predicted to go back into solution and be replaced by a proton. We note that V is not a significant species in CDI feed waters—V-insertion in Co LH was studied here solely to help understand the behavior of the Co-V LH. Considering how rapidly the cation insertion processes proceed in Fig. 3b, the Co vacancies responsible for the V-insertion mechanism may reside at the edges of LH platelets. High-rate V-insertion may also occur in smaller nanoplatelets, or arise from some other mechanism which allows for rapid V uptake. This proposed V insertion mechanism may be useful in some applications but is undesirable for CDI anion insertion electrodes. As such, the Co LH is more appealing for use as a CDI electrode.

Secondly, UEB calculations on Co_3_(OH)_6_Cl predict expulsion of Cl^−^ from the host structure (i.e. formation of Cl^−^ vacancies) at a potential more negative than −0.7 V vs. Ag/AgCl. This may contribute to anionic charge storage. However, considering the measured m/z of 17 g/mol e^−^ in Fig. 3b, OH^−^ is expected to be the dominant species responsible for the anion mechanism at these more negative potentials. We note that that at a pH of 10, the cobalt Pourbaix diagram predicts that potentials more negative than −0.6 V vs. Ag/AgCl will drive the reduction of Co(OH)_2_ to form metallic Co^56,57^, depicted by the vertical red dashed line in Fig. 4. This process is likely responsible for the anion mechanism measured under negative potentials in Fig. 3b and is consistent with the plateau observed for the Co LH measured in Fig. 3a. Extensive structural reorganization necessary to convert Co(OH)_2_ to metallic Co is expected to limit the rate of this process, in line with the slow rate of the anion-mediated process in Fig. 3b. We note that the UEB calculations we perform do not capture the conversion of Co(OH)_2_ to Co because we limited our modeling to incremental structural changes and did not evaluate complete structural reorganizations.

Although our calculations indicate that reversible anion insertion/expulsion does not occur in the Co LH under applied *negative* bias, the UEB model predicts that Cl^−^ insertion is expected to proceed in Co LH under *positive* bias as we observe in Fig. 2. Examining the chloride vacancy (*v*_*Cl*_) trace in Fig. 4, the formation energy is greater than zero at potentials more positive than −0.8 V vs. Ag/AgCl. This suggests Cl^−^ will insert into interplanar *v*_*Cl*_ sites in this potential region. However, the corresponding electron transfer (kink in trace marked by a symbol) is not predicted to occur until potentials >0.1 V vs. Ag/AgCl. Without electron transfer to balance charge with the inserting Cl^−^ anions, Coulombic repulsion is expected to limit the extent of Cl^−^ insertion into *v*_*Cl*_ sites. Once a potential >0.1 vs. Ag/AgCl is applied, electron transfer will proceed, driving additional Cl^−^ into interplanar *v*_*Cl*_ sites. This separation in the electrochemical potentials at which ion transfer and electron transfer occur has been observed in manganese oxide electrodes^40,41,53,54^ and is consistent with the irreversibility of this electrochemical mechanism, for example, requiring a 0.6 V window in Fig. 2 to undergo reversible anion insertion.

Various studies have identified an OH^–^mediated electrochemical mechanism in Co(OH)_2_ under strong basic conditions^32,58–61^. *Ex situ* Fourier transform infrared spectroscopy (FTIR) after holding at various electrochemical potentials identified that under strong basic conditions (pH ~13) a phase change from cobalt hydroxide (Co(OH)_2_) to cobalt oxyhydroxide (CoOOH) occurs at oxidizing potentials >−0.1 V vs. Ag/AgCl, which was expressed in this prior work as

3 $$Co{(OH)}_{2}+O{H}^{-}\rightleftharpoons CoOOH+{H}_{2}O+{e}^{-}$$

The formation of cobalt oxyhydroxide^61^ consistent with these observations can also be expressed as the superposition of two reactions:

4 $$Co{(OH)}_{2}+O{H}^{-}\rightleftharpoons Co{(OH)}_{3}+{e}^{-}$$

and

5 $$3Co{(OH)}_{2}+2O{H}^{-}\rightleftharpoons C{o}_{3}{O}_{4}+4{H}_{2}O+2{e}^{-}$$

Reactions 4 and 5 are predicted in cobalt Pourbaix diagrams to proceed simultaneously to form Co(OH)_3_/Co_3_O_4_ at potentials >−0.1 V vs. Ag/AgCl at a pH of 13^56,57^. This is the same potential at which reaction 3 is reported to proceed, and these reactions point generally to a transition from Co(II) to Co(III) at oxidizing potentials >−0.1 V vs. Ag/AgCl at a pH of 13. At the pH of 10 used in our work, this equilibrium potential is shifted to +0.1 V vs. Ag/AgCl. We indicate this equilibrium potential with a vertical purple dashed line in Fig. 4 labeled as Co(II)/Co(III).

The anion insertion mechanism described using the UEB construct in Fig. 4 provides additional insight into the electrochemical oxidation of Co(OH)_2_ to convert Co(II) to Co(III). Prior studies of Co(OH)_2_ electrochemistry have focused on strong basic environments, which have an excess of OH^−^. Our experimental measurements here are performed at a more neutral pH of ~10 in an excess of Cl^−^ (or Br^−^). The computational results in Fig. 4 suggest a new mechanism where Cl^−^ inserts into the interplanar space of the Co LH at an equivalent oxidizing potential to reactions 1–3. This mechanism can be expressed generally as

6 $$M{(OH)}_{2}+{A}^{-}\rightleftharpoons M{(OH)}_{2}A+{e}^{-}$$

where M is a transition metal in a LH structure (e.g. Co, Ni, Mn, etc.) and A^−^ is an anion (e.g. Cl^−^, Br^−^, OH^−^, etc.). This mechanism with M = Co and A^−^ = Cl^−^ (reaction 2) yields an expected m/z of 34.5 g/mol e^−^, consistent with the m/z > 30 g/mol e^−^ measured using EQCM in Fig. 2. The value lower than 34.5 may indicate that the OH^−^ anion is also contributing to these electrochemical processes. High energy X-ray diffraction data further corroborates that anions insert into the interplanar space, as described in the following section.

### *In Situ* Measurements to Confirm Mechanistic Understanding

**Figure 5:**
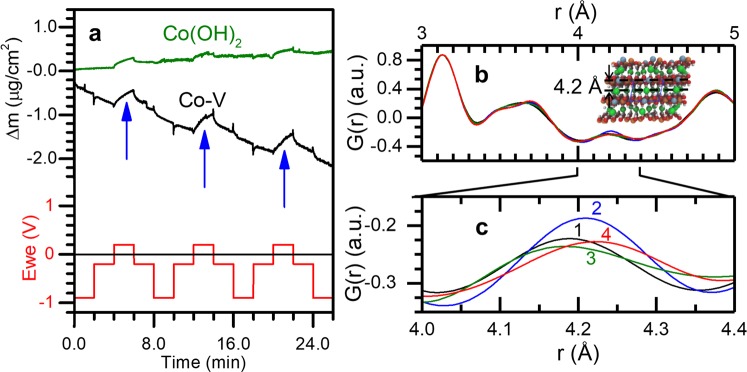
EQCM and PDF during oxidation of Co-V and Co LHs. (**a**) EQCM cycling of Co-V and Co LH reveals anion-based mechanism under applied positive bias (+0.2 V vs. Ag/AgCl). This is corroborated by (**b**) *in situ* PDF analysis of Co-V showing (**c**) changes in the pair distribution function (G(r)) intensity when switching from (1) −0.2 V vs. Ag/AgCl to (2) +0.2 V vs. Ag/AgCl, and back to (3) −0.2 V vs. Ag/AgCl consistent with reversible insertion of interplanar anions. Little change in G(r) was observed under (4) −0.9 V vs. Ag/AgCl.

Based on the UEB modeling in Fig. 4 and the supporting literature described above, we expect that anion insertion will occur in the Co LH as Co(II) converts to Co(III) under applied positive bias >0.1 V vs. Ag/AgCl. This mechanism is also expected to manifest in Co LH domains of the Co-V LH. To evaluate this, we examine *in situ* EQCM, HE-XRD, and XAS data for LH platelets cycled in potential loops of {−0.9 V, −0.2 V, +0.2 V, −0.2 V}. We first examine the EQCM data in Fig. 5a. We attribute the overall decrease in mass of the Co-V LDH to delamination of LH platelets as well as the favorable dissolution of V as predicted from UEB modeling. We again note that the Co LH is more appealing for CDI electrodes and a polymeric binder could be used to mitigate delamination. The potential-dependent mass changes for both the Co-V and Co LH samples in Fig. 5a are consistent with anion insertion. We observe a mass gain under positive bias (+0.2 V) highlighted with blue arrows as anions are drawn into the interplanar space of the LHs, and a mass loss under reverse bias as anions leave the LHs. The similarity in results for the Co-V and Co LH samples in Fig. 5a supports the prediction that anion insertion takes place in Co LH domains of the Co-V LH sample. These results are also consistent with the EQCM results for the Co LH presented in Fig. 2a.

We also performed *in situ* HE-XRD measurements using a custom electrochemical cell^62^ to elucidate the structural changes during electrochemical cycling. Depicted in Fig. 5b are PDFs calculated from *in situ* HE-XRD data during electrochemical operation. In these traces, we observe a peak at a pair distance of r = 4.2 Å which corresponds to the distance between interplanar anions and metal ions in the hydroxide sheets. Twice this distance corresponds to the interplanar spacing, here 8.4 Å. This is larger than the interplanar distance of ~8 Å observed in Fig. 1d. We attribute the increase in interplanar distance here to the use of the Br^−^ anion for *in situ* measurements to enhance diffraction from a heavier interplanar anion. The diameter of Br^−^ is 3.92 Å, while the diameter of Cl^−^ is 3.62 Å^13^. We interpret an increase in G(r) intensity at r ≈ 4.2 Å in Fig. 5b,c as an increase in the number of interplanar anions. In Fig. 5c, G(r) intensity increases at r ≈ 4.2 Å when switching from −0.2 V (trace 1) to +0.2 V (trace 2) vs. Ag/AgCl, indicating the incorporation of anions at +0.2 V. G(r) intensity then decreases at r ≈ 4.2 Å when switching from +0.2 V (trace 2) to −0.2 V (trace 3) vs. Ag/AgCl, indicating the release of interplanar anions. Furthermore, we note little change in the G(r) intensity at r ≈ 4.2 Å when switching from −0.2 V (trace 3) to −0.9 V (trace 4) vs. Ag/AgCl, further corroborating our conclusion that interplanar anions do not leave the structure under negative bias. These HE-XRD results are consistent with the EQCM results presented above and suggest that reaction 2 is occurring in the Co LH.

**Figure 6:**
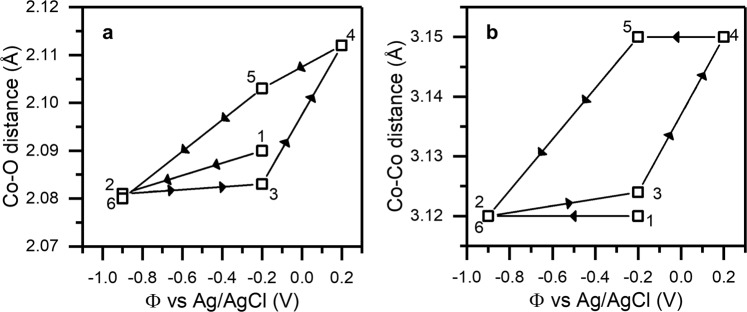
EXAFS of Co-V LH under oxidizing potentials. Changes in (**a**) Co-O distance and (**b**) Co-Co distance from *in situ* XAS measurements during potentiostatic electrochemical operation. The bond-length changes we observe are consistent with the formation of charged *v*_*Co*_ sites in the Co_3_(OH)_6_Cl structure.

While EQCM and PDF analysis provide strong support of electrochemical anion insertion in Co LH domains of the Co-V LDH sample, extended X-ray absorption fine structure (EXAFS) measurements on the Co-V LDH over longer timescales suggests a competing Co dissolution mechanism. See supporting information for details on EXAFS experimentation. The results of steady-state EXAFS measurements are depicted in Fig. 6, which indicate that the Co-Co/V nearest neighbor distance increases from 3.12 Å to 3.15 Å, and the Co-O nearest neighbor distances increase from 2.08 Å to 2.11 Å when switching from −0.2 V to +0.2 V vs. Ag/AgCl. While *ab initio* modeling predicts minimal changes in the Co-Co and Co-O distances due to Cl^−^ insertion, these structural changes are in line with the formation of *v*_*Co*_ at this potential. *Ab initio* calculations for the formation of Co vacancies near V dopants (modeled in the Co_3_V(OH)_8_Cl structure) predict these defects to occur at potentials > +0.2 V vs. Ag/AgCl in Fig. 3d, leading to an increase the average Co-Co distance from 3.01 Å in the perfect structure to 3.14 Å in a charged *v*_*Co*_ structure, and an increase in the average Co-O distance from 2.06 Å in the perfect structure to 2.09 Å in a charged *v*_*Co*_ structure. However, we do not observe indicators for this mechanism in EQCM or PDF measurements. The formation of *v*_*Co*_ likely proceeds at too slow of a rate to be observed during relatively fast EQCM and PDF measurements (minutes timescale), but allowing for observation at the timescale of our EXAFS measurement (hours timescale).

We also note that we do not observe changes in Co oxidation state in the X-ray absorption near-edge spectrum (XANES) at various applied biases (Fig. S10). The Co K-edge amplitude (Fig. S11) and Co K-edge real space fitting results (Fig. S12) are also provided in ESI Section I. While a change in the charge state of Co ions is not requisite during charging of the Co LH for anion insertion^63^, a charge state change is expected for the formation of metallic Co under applied negative bias (−0.2 V), as described above. The absence of zero-valent Co in the XANES data under negative bias may suggest that the thermodynamic description laid out in the Co Pourbaix diagram does not fully capture the processes occurring under negative bias, or that only a small fraction of metallic Co forms during EXAFS measurements. More importantly, these findings suggest that additional work is needed to further understand the electrochemical processes in these promising materials.

## Conclusions

In this work, we combine *ab initio* modeling and *in situ* experimental studies to identify a previously undescribed mechanism of electrochemical anion insertion in layered cobalt hydroxides. We establish the electrochemical mechanisms occurring in Co and Co-V LH in aqueous electrolyte under both negative and positive applied bias. Under applied negative bias, we identify mechanisms for cation exchange (e.g. V insertion on Co site) and phase change (i.e. OH^−^ removal to form metallic Co). Under positive bias, we observe electrochemical anion insertion—anions are drawn into the interplanar spaces of the LHs under positive bias and driven out under reverse bias. We also observe evidence of slow dissolution of Co under positive bias, and of delamination during electrochemical cycling. Our data suggests that Co LH enables anion insertion electrochemistry within the potential limits of water stability, while larger potentials are needed for anion insertion in other Co-containing LHs (*e*.*g*. Co_3_V LDH). With further refinement to prevent dissolution and delamination, enhance ion insertion rates, and improve stability, LHs based on this work could be designed to be paired with a cation insertion electrode material and used for energy efficient electrochemical desalination.

The mechanistic understanding of layered hydroxide electrochemistry we establish here also has implications in a range of other fields. While LH nanoplatelets have been studied for battery^36,37^ and supercapacitor^32–35^ applications, the observed charge storage has been attributed to mechanisms of OH^−^ reaction at metal centers (i.e. M(OH)_2_/MOOH)^32,61^ and/or cation insertion^36^. The anion-insertion electrochemical mechanism we describe here has not been realized, and will impact system design for energy storage devices using LHs. Conventional (e.g. lithium ion) battery designs rely on cation insertion, and if the anion insertion mechanism we identify proves to be active in other LHs, this will present a range of opportunities for developing novel battery and supercapacitor systems. The viability of pairing anion insertion and cation insertion materials in a single device could be explored, along with the viability of pairing two LHs to make purely anion-based intercalation batteries for energy storage. New electrolytes could be developed with enhanced anion mobility or which contain anions tailored for rapid insertion into the interplanar spacing of LHs. Furthermore, the electrochemically induced anion release mechanism we describe could impact prospects for drug delivery using LHs^26^. For example, a system could be envisioned by which a (photo)electrochemical stimulus is used for spatially-controlled delivery of thereputic polyanions, including DNA or RNA fragments.

## Supplementary information

Supplementary

## References

[1] Buluswar, S., Friedman, Z., Mehta, P., Mitra, S. & Sathre, R. *50 Breakthroughs - Critical scientific and technological advances needed for sustainable gobal development*. (LIGTT, Institute of Globally Transformative Technologies, Lawrence Berkeley National Lab, 2014).

[2] Zhao R, Porada S, Biesheuvel PM, Van der Wal A (2013). Energy consumption in membrane capacitive deionization for different water recoveries and flow rates, and comparison with reverse osmosis. Desalination.

[3] Pasta M, Wessells CD, Cui Y, La Mantia F, Mantia FL (2012). A desalination battery. Nano Lett..

[4] Suss ME, Presser V (2018). Water Desalination with Energy Storage ElectrodeMaterials. Joule.

[5] Porada S, Shrivastava A, Bukowska P, Biesheuvel PM, Smith KC (2017). Nickel Hexacyanoferrate Electrodes for Continuous Cation Intercalation Desalination of Brackish Water. Electrochim. Acta.

[6] Lee J, Kim S, Yoon J (2017). Rocking Chair Desalination Battery Based on Prussian Blue Electrodes. ACS Omega.

[7] Kim T, Gorski CA, Logan BE (2017). Low Energy Desalination Using Battery Electrode Deionization. Environ. Sci. Technol. Lett..

[8] Hickner MA (2017). Strategies for Developing New Anion Exchange Membranes and Electrode Ionomers. Electrochem. Soc. Interface.

[9] Bard, A. J. & Faulkner, L. R. *Electrochemical Methods: Fundamentals and Applications*. (John Wiley & Sons). doi:0471043729 (2000).

[10] Katan T, Szpak S, Bennion DN (1974). Silver/Silver Chloride Electrodes: Surface Morphology on Charging and Discharging. J. Electrochem. Soc..

[11] Droog JMM, Huisman F (1980). Electrochemical formation and reduction of silver oxides in alkaline media. J. Electroanal. Chem..

[12] Fabrega J, Luoma SN, Tyler CR, Galloway TS, Lead JR (2011). Silver nanoparticles: Behaviour and effects in the aquatic environment. Environ. Int..

[13] *CRC Handbook of Chemistry and Physics* (*Internet Version 2017*). (CRC Press/Taylor & Francis, 2017).

[14] Morita M, Komaguchi K, Tsutsumi H, Matsuda Y (1992). Electrosynthesis of poly(p-phenylene) films and their application to the electrodes of rechargeable batteries. Electrochim. Acta.

[15] Pruss A, Beck F (1984). Reversible electrochemical insertion of anions in poly-p-phenylene from aqueous electrolytes. J. Electroanal. Chem..

[16] Bird CL, Kuhn AT (1981). Electrochemistry of the viologens. Chem. Soc. Rev..

[17] Mefford JT, Hardin WG, Dai S, Johnston KP, Stevenson KJ (2014). Anion charge storage through oxygen intercalation in LaMnO_3_ perovskite pseudocapacitor electrodes. Nat. Mater..

[18] Chen F (2017). Dual-ions electrochemical deionization: A desalination generator. Energy Environ. Sci..

[19] Slater MD, Kim D, Lee E, Johnson CS (2013). Sodium-ion batteries. Adv. Funct. Mater..

[20] Nitta N, Wu F, Lee JT, Yushin G (2015). Li-ion battery materials: Present and future. Mater. Today.

[21] Saha P, Kanchan M, Velikokhatnyi OI (2014). Progress in Materials Science Rechargeable magnesium battery: Current status and key challenges for the future. Prog. Mater. Sci..

[22] Hwang J-Y, Myung S-T, Sun Y-K (2017). Sodium-ion batteries: present and future. Chem. Soc. Rev..

[23] Miyata S (1983). Anion-Exchange Properties of Hydrotalcite-Like Compounds. Clays Clay Miner..

[24] Khan AI, O’Hare D (2002). Intercalation chemistry of layered double hydroxides: recent developments and applications. J. Mater. Chem..

[25] Liu Z, Ma R, Osada M, Takada K, Sasaki T (2005). Selective and controlled synthesis of α- and β-cobalt hydroxides in highly developed hexagonal platelets. J. Am. Chem. Soc..

[26] Choy J-H, Kwak S-Y, Jeong Y-J, Park J (2000). Inorganic Layered Double Hydroxides as Nonviral Vectors. Angew. Chemie.

[27] Wang Q, Ohare D (2012). Recent advances in the synthesis and application of layered double hydroxide (LDH) nanosheets. Chem. Rev..

[28] Regier T, Wei F, Dai H (2013). An Advanced Ni−Fe Layered Double Hydroxide Electrocatalyst for water oxidation. J. Am. Chem. Soc..

[29] Song F, Hu X (2014). Exfoliation of layered double hydroxides for enhanced oxygen evolution catalysis. Nat. Commun..

[30] Maeda K, Ishimaki K, Tokunaga Y, Lu D, Eguchi M (2016). Modification of Wide-Band-Gap Oxide Semiconductors with Cobalt Hydroxide Nanoclusters for Visible-LightWater Oxidation. Angew. Chemie - Int. Ed..

[31] Zhao Y (2015). Layered Double Hydroxide Nanostructured Photocatalysts for Renewable Energy Production. Adv. Energy Mater..

[32] Wang Y, Yang W, Yang J (2007). A Co–Al Layered Double Hydroxides Nanosheets Thin-Film Electrode. Electrochem. Solid-State Lett..

[33] Chen H, Hu L, Chen M, Yan Y, Wu L (2014). Nickel-cobalt layered double hydroxide nanosheets for high-performance supercapacitor electrode materials. Adv. Funct. Mater..

[34] Xiao Y (2017). Ultrahigh energy density and stable supercapacitor with 2D NiCoAl Layered double hydroxide. Electrochim. Acta.

[35] Liu X (2016). Ultrahigh-rate-capability of a layered double hydroxide supercapacitor based on a self-generated electrolyte reservoir. J. Mater. Chem. A.

[36] Latorre-Sanchez M (2012). The synthesis of a hybrid graphene-nickel/manganese mixed oxide and its performance in lithium-ion batteries. Carbon N. Y..

[37] Gong M (2014). Ultrafast high-capacity NiZn battery with NiAlCo-layered double hydroxide. Energy Environ. Sci..

[38] Lei X (2014). Three-dimensional NiAl-mixed metal oxide film: Preparation and capacitive deionization performances. RSC Adv..

[39] Ren Q (2018). Calcined mgal-layered double hydroxide/graphene hybrids for capacitive deionization. Ind. Eng. Chem. Res..

[40] Young MJ, Holder AM, George SM, Musgrave CB (2015). Charge Storage in Cation Incorporated α-MnO_2_. Chem. Mater..

[41] Young MJ, Schnabel H-D, Holder AM, George SM, Musgrave CB (2016). Band Diagram and Rate Analysis of Thin Film Spinel LiMn_2_O_4_ Formed by Electrochemical Conversion of ALD-Grown MnO. Adv. Funct. Mater..

[42] Gastuche MC, Brown G, Mortland MM (1967). Mixed Magnesium-Aluminium Hydroxides. Clay Miner..

[43] Brown G, Gastuche MC (1967). Mixed Magnesium-Aluminium Hydroxides II. Structure and Structural Chemistry of Synthetic Hydroxycarbonates and Related Minerals and Compounds. Clay Miner..

[44] Miyata S (1975). The Syntheses of Hydrotalcite-Like Compounds and Their Structures and Physico-Chemical Properties I: The Systems Mg^2+^-Al^3+^-NO_3_^−^, -Cl^−^, -ClO_4_^−^, Ni^2+^ and Zn^2+^. Clays Clay Miner..

[45] Xu ZP, Stevenson G, Lu C-Q, Lu GQM (2006). Dispersion and size control of layered double hydroxide nanoparticles in aqueous solutions. J. Phys. Chem. B.

[46] Vialat P (2014). High-performing monometallic cobalt layered double hydroxide supercapacitor with defined local structure. Adv. Funct. Mater..

[47] Song F, Hu X (2014). Ultrathin cobalt-manganese layered double hydroxide is an efficient oxygen evolution catalyst. J. Am. Chem. Soc..

[48] Ruffo R, Wessells C, Huggins RA, Cui Y (2009). Electrochemical behavior of LiCoO_2_ as aqueous lithium-ion battery electrodes. Electrochem. commun..

[49] Guo X, Zhang F, Evans DG, Duan X (2010). Layered double hydroxide films: synthesis, properties and applications. Chem. Commun. (Camb)..

[50] Petkov V (2008). Nanostructure by high-energy X-ray diffraction. Mater. Today.

[51] Billinge, S. J. L. & Kanatzidis, M. G. Beyond crystallography: the study of disorder, nanocrystallinity and crystallographically challenged materials with pair distribution functions. *Chem*. *Commun*. 749–760, 10.1039/b309577k (2004). 10.1039/b309577k 15045050

[52] Keen DA, McGreevy RL (1990). Structural modelling of glasses using reverse Monte Carlo simulation. Nature.

[53] Wallas JM, Young MJ, Sun H, George SM (2018). Efficient Capacitive Deionization Using Thin Film Sodium Manganese Oxide. J. Electrochem. Soc..

[54] Young MJ, Holder AM, Musgrave CB (2018). The Unified Electrochemical Band Diagram Framework: Understanding the Driving Forces of Materials Electrochemistry. Adv. Funct. Mater..

[55] Islam MS, Fisher CaJ (2014). Lithium and sodium battery cathode materials: computational insights into voltage, diffusion and nanostructural properties. Chem. Soc. Rev..

[56] Pourbaix, M. *Atlas of Electrochemical Equilibria in Aqueous Solutions*. (National Association of Corrosion Engineers, 1974).

[57] Bloor LG, Molina PI, Symes MD, Cronin L (2014). Low pH electrolytic water splitting using earth-abundant metastable catalysts that self-assemble *in situ*. J. Am. Chem. Soc..

[58] Lin C (1998). Characterization of Sol-Gel-Derived Cobalt Oxide Xerogels as Electrochemical Capacitors. J. Electrochem. Soc..

[59] Scavetta E, Ballarin B, Gazzano M, Tonelli D (2009). Electrochemical behaviour of thin films of Co/Al layered double hydroxide prepared by electrodeposition. Electrochim. Acta.

[60] Švegl F, Orel B, Hutchins MG, Kalcher K (1996). Structural and Spectroelectrochemical Investigations of Sol-Gel Derived Electrochromic Spinel Co3O4 Films. J. Electrochem. Soc..

[61] Boggio R, Carugati A, Trasatti S (1987). Electrochemical surface properties of Co3O4 electrodes. J. Appl. Electrochem..

[62] Young MJ, Bedford NM, Jiang N, Lin D, Dai L (2017). *In situ* electrochemical high-energy X-ray diffraction using a capillary working electrode cell geometry. J. Synchrotron Radiat..

[63] Raebiger H, Lany S, Zunger A (2008). Charge self-regulation upon changing the oxidation state of transition metals in insulators. Nature.

