# Supplementary material for: Discovery of Anion Insertion Electrochemistry in Layered Hydroxide Nanomaterials
Source: Sci Rep. 2019 Feb 21;9:2462. doi: 10.1038/s41598-019-39052-1 (PMC6384940; doi:10.1038/s41598-019-39052-1)
Supplement: Supplementary file 1 — Supplementary [file 41598_2019_39052_MOESM1_ESM.pdf]

Discovery of Anion Insertion Electrochemistry in Layered Hydroxide Nanomaterials

Matthias J. Young[1,2,3,4,*], Tatyana Kiryutina[2], Nicholas M. Bedford[2,5,*], Taylor J. Woehl[2,6], Carlo U. Segre[7]

[1] Applied Materials Division, Argonne National Laboratory, Argonne, Illinois, 60439, United States

[2] Applied Chemicals and Materials Division, National Institute of Standards and Technology, Boulder, Colorado, 80305, United States

[3] Department of Biomedical, Biological, and Chemical Engineering, University of Missouri, Columbia, MO, 65211, United States

[4] Department of Chemistry, University of Missouri, Columbia, MO, 65211, United States

[5] School of Chemical Engineering, University of New South Wales, Sydney, NSW 2052, Australia

[6] Department of Chemical and Biomolecular Engineering, University of Maryland, College Park, Maryland, 20742, United States

[7] Department of Physics, Illinois Institute of Technology, Chicago, Illinois, 60616, United States

[*]Email: matthias.young@missouri.edu or n.bedford@unsw.edu.au

Electronic Supplementary Information

A. Layered Hydroxide Synthesis

Layered hydroxide nanoparticles were synthesized using coprecipitation.[1] Salt solutions for nanoparticle synthesis were prepared using chloride salts for Co: cobalt(II) chloride (anhydrous, 99.7%, Alfa Aesar), Cr: chromium(III) chloride hexahydrate (98%, Alfa Aesar), V: vanadium(III) chloride (anhydrous, 99%, Alfa Aesar), Al: aluminum chloride hexahydrate (99%, Alfa Aesar), and Mg: magnesium chloride hexahydrate (ACS Grade, Ampresco). For LDHs, 1.5 mmol of M^{3+} salt was combined with 0.5 mmol of M^{2+} salt in 5 mL of deionized (DI) water (18.2 MΩ, Millipore Direct-Q) to make a salt solution. For Co LH, 2 mmol of salt was dissolved in 5 mL of DI water to make a salt solution.

20 mL of 0.15 M NaOH (ACS Grade, EMD Millipore) was purged for > 15 minutes under argon (Ar, UHP, General Air Service and Supply) atmosphere in a 3-neck flask at room temperature (~ 20°C). 5 mL of salt solution (see above) was rapidly injected into the NaOH solution, and held under Ar atmosphere for > 15 minutes to seed nanoparticle formation. The nanoparticles were rinsed three times by: (1) centrifuging the ~ 25 mL nanoparticle suspension for 3 minutes at 7000

Official contribution of the National Institute of Standards and Technology; not subject to copyright in the United States.

The submitted manuscript has been created by UChicago Argonne, LLC, Operator of Argonne National Laboratory ("Argonne"). Argonne, a U.S. Department of Energy Office of Science laboratory, is operated under Contract No. DE-AC02-06CH11357. The U.S. Government retains for itself, and others acting on its behalf, a paid-up nonexclusive, irrevocable worldwide license in said article to reproduce, prepare derivative works, distribute copies to the public, and perform publicly and display publicly, by or on behalf of the Government.

rpms (2) pouring off the supernatant, and (3) resuspending the nanoparticles in DI water to return the total volume of the suspension to ~25 mL. The resulting aqueous suspensions contained ~ 3µg/µL of nanoparticles. Following this synthesis procedure, the nanoparticles were either aged using hydrothermal treatment at 100°C for 4 hours[2], or at room temperature for at least one week.

B. Electron Microscopy

High resolution ADF STEM imaging was performed on a JEOL ARM200F with a Schottky field emission source operating at 200 kV. Samples were prepared by diluting LH suspensions to 1% in DI water and drop casting 5 µL of sample onto ultrathin carbon coated lacey carbon grids (Ted Pella, USA) and plasma cleaning in a 97% hydrogen, 3% oxygen plasma (Fischione model 1070 plasma cleaner) for 1 minute directly prior to imaging. ADF STEM images were acquired under relatively low dose conditions to avoid damage of the LDH particles. The beam current was ~20 pA and the pixel dwell time was 25 µs. The ADF detector inner collection angle was ~70 mrad.

STEM-EDS elemental mapping was performed on an FEI Talos F200X TEM operating at 200 kV with a Schottky field emission source and Bruker ChemiSTEM EDS system. Pixel intensities in the EDS maps for Mg, Al, and O were computed by integrating the $L\alpha_1$ peaks for each element.

STEM imaging on Co LH were performed in a similar manner using a TalosTM operating at 200 kV. Presented in Figure S1 is an ADF STEM micrograph of Co LH nanoplatelets synthesized without a trivalent metal center. These nanoplatelets are similar in shape to the LDH structures, but are only ~ 10 nm in diameter rather than ~ 100 nm as observed for the LDH compositions. These smaller $Co(OH)_2$ nanoplatelets are consistent with the explanation above describing the presence of $Co(OH)_2$ in the Co-V nanoplatelet sample.

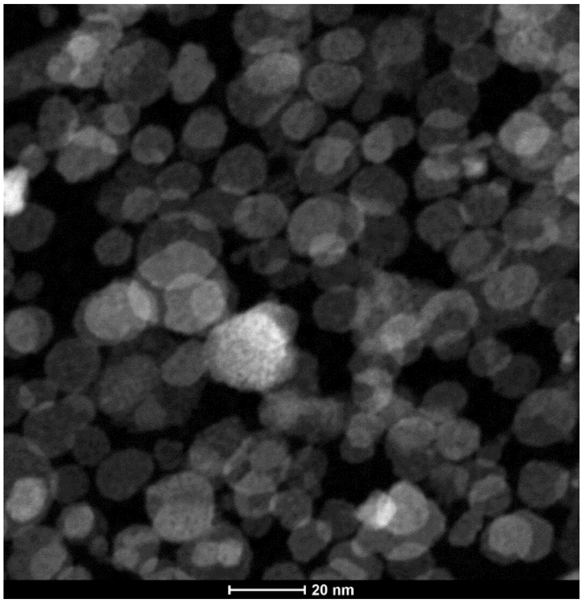

Figure S1 *STEM of Co LH.* Micrograph of Co LH shows monodispersed nanoplatelets of ~ 10 nm diameter. An order of magnitude smaller than the LDH nanoplatelets.

Presented in Figure S2 is an ADF STEM image of the Co-V LH. The nanoplatelet morphology and size we observe is consistent with the LH structure, and agrees closely with the STEM results for Mg-Al and Co-Al LHs presented in Figure 1. An exemplary energy dispersive x-ray spectroscopy (EDS) measurement of a Co-V nanoplatelet is presented in Figure S3. This EDS spectrum clearly shows the presence of both Co and V in these nanoplatelets. The signal for Cu arises from the TEM grid. EDS quantitative analysis identified an average Co:V ratio of 3.3±0.4 by sampling seven particles. One measurement indicated a Co:V ratio of 5.8, but was a statistical outlier and was excluded from the calculation of this average value.

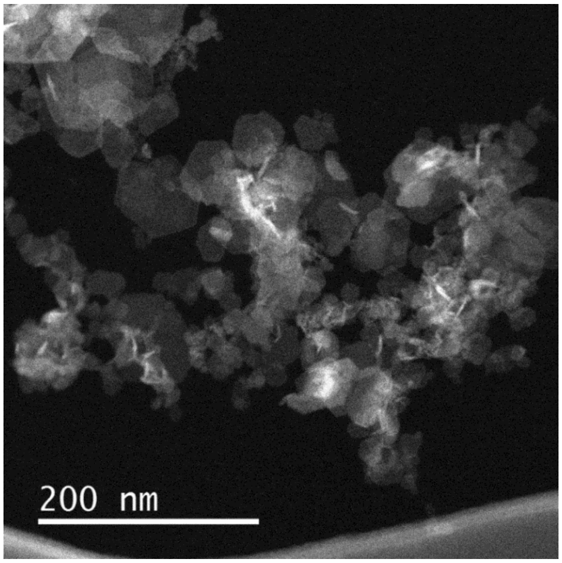

Figure S2 *STEM of Co-V LDH.* Micrograph of Co-V LDH nanoplatelets shows analogous morphology and particle size to Mg-Al and Co-Al nanoplatelets.

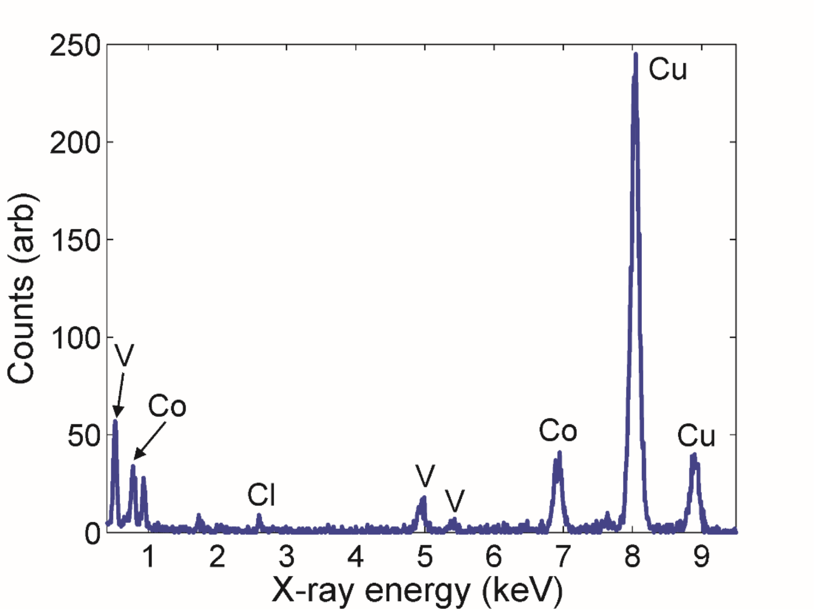

Figure S3 *EDS spectrum of Co-V LDH nanoplatelets.* EDS indicates presence of Co and V in a 3:1 ratio in the Co-V LDH sample. Also observe interplanar Cl, and Cu from TEM grid.

Interestingly, the Co:V ratio of ~3 determined by STEM-EDS is much smaller than the value of 48 determined by ICP-OES following digestion. We attribute this discrepancy to a bias in the STEM-EDS analysis toward larger, structurally defined nanoplatelets. During STEM of the Co-V we did observe agglomerates of smaller nanoplatelets which were not stable under the electron beam and could not be characterized by EDS. We suspect that these smaller nanoplatelets were $Co(OH)_2$, leading to a lower average concentration of V when the entire sample is digested.

C. High Energy X-ray Diffraction (HE-XRD)

HE-XRD was performed both *in situ* and *ex situ* at the 6-ID-D beamline at the Advanced Photon Source (APS) at Argonne National Lab using 100 keV irradiaton. Supplemental *ex situ* HE-XRD measurements of Mg_2Al and Co LHs were performed at 11-ID-B and 11-ID-D beamlines using 58.65 and 105 keV irradiaton, respectively. All measurements were performed in a transmission configuration with the sample-to-detector distance set to maximize the sampled q-range.

For HE-XRD characterization, LH nanoparticle suspensions were frozen overnight at -80°C, then lyophilized (VirTis, Benchtop K) to produce bulk nanoparticle powders. For *ex situ* measurements, these powders were crushed and ground into fine particles, then loaded into Kapton capillaries (1mm dia., Cole-Palmer) and measured in an automated fashion with a multi-sample stage. *In situ* measurements during electrochemical operation were performed using a capillary working electrode cell geometry as described elsewhere[3] Here we use conductive granular activated carbon (Norit GAC 400 M-1746) as an electrode support inside of the carbon fiber capillary working electrode, and we add lyophilized LH powder on top of the GAC. The X-ray beam position was adjusted to the GAC/active material interface to probe active LH material in electrical contact with the conductive carbon support.

Background correction, conversion to total structure functions, and Fourier transformation to produce atomic pair distribution functions (PDFs) was performed using PDFgetX3.[4] Diffracted X-ray intensity, i(Q), reduced total scattering factor, F(Q), and pair distribution function, G(r), for the Mg-Al LDH are plotted in Figure S4a. Reference peak locations for the Mg-Al LDH structure[5] are shown in blue and the $Co(OH)_2$ LH structure[6] are shown in red, as simulated using the RIETAN-FP·VENUS[7] package as implemented in VESTA.[8] Using starting structures from *ab initio* modeling (see below), a first-pass structural fit was performed on a unit cell structure using PDFgui with optimization of lattice vectors only. The output structures from PDFgui were expanded into supercells with 40Å minimum dimensions, and used as starting structures for reverse monte carlo (RMC) modeling. RMC modeling was performed for *ex situ* samples using the fullrmc python package.[9] The fitted RMC structure for the Mg-Al LDH is shown in Figure S4b. Additional plots of i(Q), F(Q), and G(r) for the Co, Co-V, Co-Al, and Co-Cr LHs are presented in Figure S5a-d, respectively.

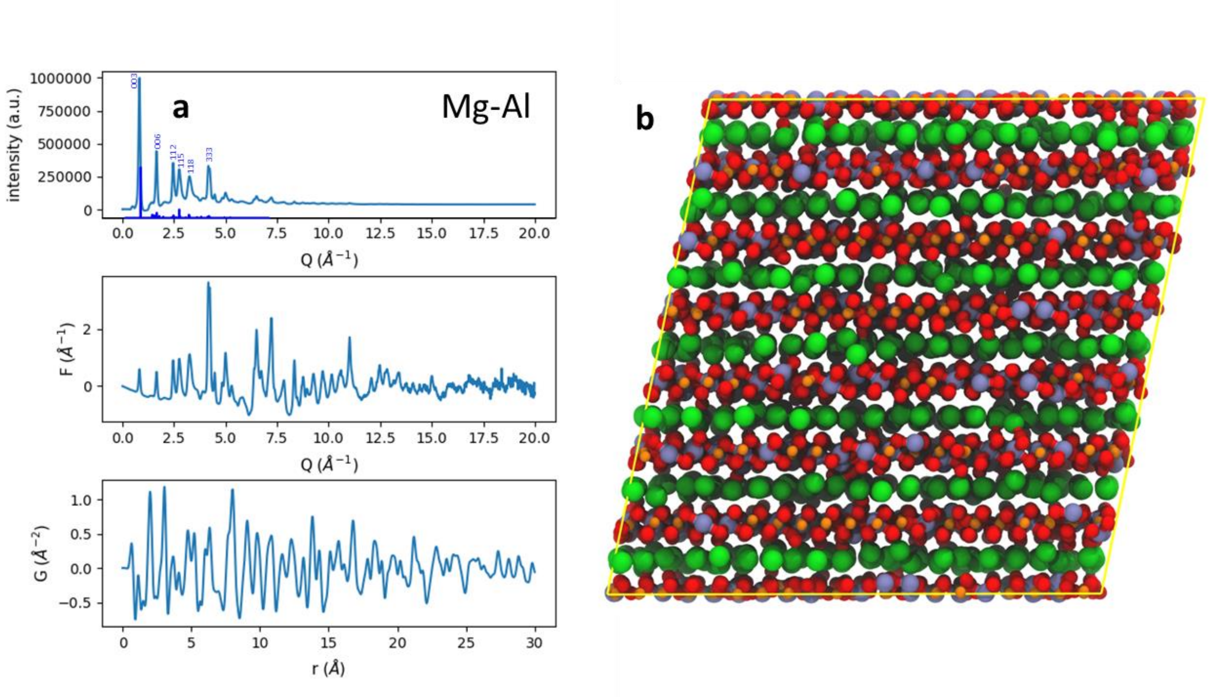

Figure S4 *PDF and RMC on Mg-Al LDH.* (a) Pair distribution function calculation including raw diffracted X-ray intensity, i(Q), with reference peak locations for Mg-Al LDH[5] (blue), reduced total scattering factor, F(Q), and pair distribution function, G(r), for the Mg-Al LDH. (b) Final RMC periodic cell structure for the Mg-Al LDH fit to the PDF data in (a).

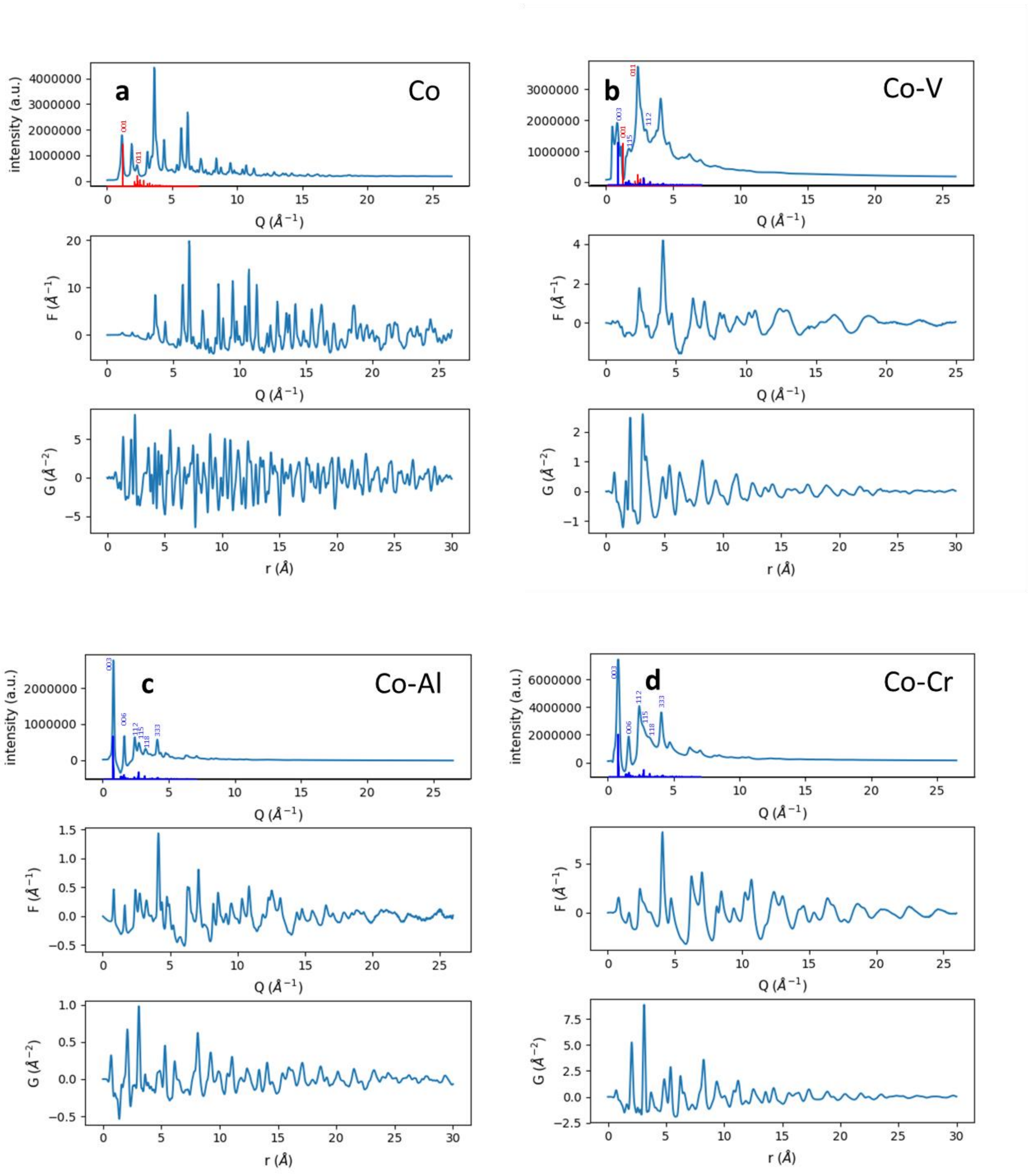

Figure S5 *PDF analysis on Co LHs.* Raw diffracted X-ray intensity, i(Q), with reference peak locations for Mg-Al LDH[5] (blue) and $Co(OH)_2$[6] (red), reduced total scattering factor, F(Q), and pair distribution function, G(r) for (a) Co, (b) Co-V, (c) Co-Al, and (d) Co-Cr LHs.

D. Ab Initio Modeling

LH electrochemical thermodynamics were modeled using the unified electrochemical band-diagram (UEB) framework, as described in prior work.[10–12] We model various point defects in LH structures including substitutions, vacancies, and interstitials, with primary focus on the electrochemically active $Co_3(OH)_6Cl$ and $Co_2V(OH)_6Cl$ structures and Cl insertion/removal. Total energy calculations are performed for perfect and (charged) defect structures using density functional theory (DFT) and the projector augmented-wave (PAW)[13] method as implemented in the Vienna Ab initio Simulation Package (VASP)[14–16]. Chemically relevant cobalt 3d and 4s, vanadium 3p, 3d, and 4s, chlorine 3s and 3p, oxygen 2s and 2p, and hydrogen 1s electrons are calculated explicitly using PAWs, while pseudopotentials describe core electrons. A fully automatic Γ-centered Monkhorst-Pack K-point mesh was generated for all structures using VASP with l=14.

We employ the modified Heyd-Scuseria-Ernzerhof (HSEsol)[17] range-separated functional for charged defect calculations to correct for self-interaction error in defect calculations.[18,19] We carry out multi-step ionic and cell-shape relaxations as implemented in the *pylada* python module. We account for pH and applied bias in formation energies calculations by using the hydroxide-forming limit and setting the chemical potential of H based on the pH as described previously.[10] Here, we use bulk crystal hydroxide structures instead of individual hydroxide molecules for reference calculations, and we note a sign correction in the applied bias as compared with this prior work.[10] All calculations are performed using a surface description by accounting for band bending at the electrode surface. For this, we assume the Nernstian relationship $V = V_{PZC} + 0.059(pH_{PZC} - pH)$, as described previously,[10,20] and use a pH_{PZC} value of 11.4 based on the PZC reported for $Co(OH)_2$.[21]

We use the idealized 2:1 M^{2+}-M^{3+} molar ratio LDH structure depicted in Figure 1e with a honeycomb metal configuration based on the 2:1 Mg-Al LDH structure [22] as depicted in Figure S6a in the SM. We align the band edges of the material within an electrochemical reference frame using work function calculations[23] for the dominant {100} surface [24] which is perpendicular to the LH planes as depicted in Figure S6b. We employ ≥ 17 Å thick slabs, and ≥ 15 Å vacuum space, with ionic relaxation on all atoms > 5Å from the center plane of the slabs. We calculate the potential difference between vacuum and the bulk material using the Purdew-Burke-Ernzerhof (PBEsol)[25] functional and correct the band edge positions at the HSEsol level in a similar fashion to work correcting band edge energies using quasiparticle calculations.[26]

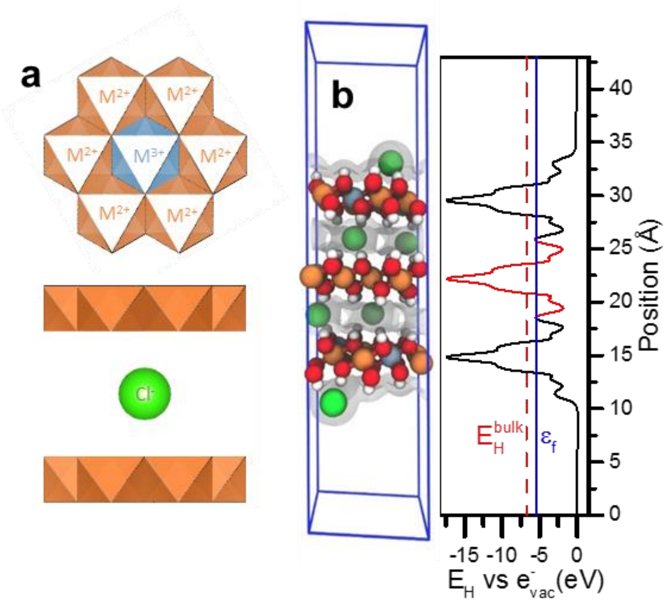

Figure S6 *Structural details for work function calculations.* (a) Honeycomb orientation of metal centers in hydroxide planes based on $Mg_2Al(OH)_6Cl$ structure and (b) plane-averaged local Hartree potential in bulk (E_H^{bulk}) vs. calculated fermi energy (ε_f).

E. *Ultra-violet Visible Spectroscopy (UV-Vis)*

UV-Vis (Cary 8454, Agilent Technologies) was performed between 230 nm and 900 nm in polystyrene disposable cuvettes (1 cm path length, PLASTIBRAND). 100 µL of nanoparticle suspension was diluted in 2 mL of DI water, and a DI water blank was measured before each sample. Tauc plot analysis was performed for direct allowed transitions (r = ½).

Presented in Figure S7 is a Tauc plot of UV-Vis data collected on aqueous LH suspensions. Tauc analysis is performed for direct allowed transitions. The band gap of each layered hydroxide is determined by extrapolating the linear portion of the trace to the intersection of $(\alpha h\nu)^2=0$. Band gaps are calculated to be 4.4 eV (Co-Al), 4.4 eV (Co-Cr), 2.7 eV (Co-V), and 3.7 eV (Co). No absorption was observed for Mg_2Al for the range of photon energies examined by UV-Vis, suggesting a band gap > 5.3 eV. We use the linear region > 4 eV for determination of the Co LH band gap, and attribute the lower-energy absorption to defect states. For the Co-V trace we observe two linear regions: one between 2.5 and 3.7 eV, and one between 4 eV and 5.3 eV. The lower-energy linear region of the Co-V trace is used for band gap determination. Using the higher energy region, we calculate a bandgap of ~3.5 eV. Similarly, a minority absorption is also observed at lower energies for Co-Al and Co-Cr corresponding to a band gap of ~ 3.5 eV. A 3.5 eV band gap agrees closely with the band gap we determine for the Co LH ($Co(OH)_2$). We suspect that the secondary absorption observed for each of the binary LDHs arises from local regions of $Co(OH)_2$ present in these compositions.

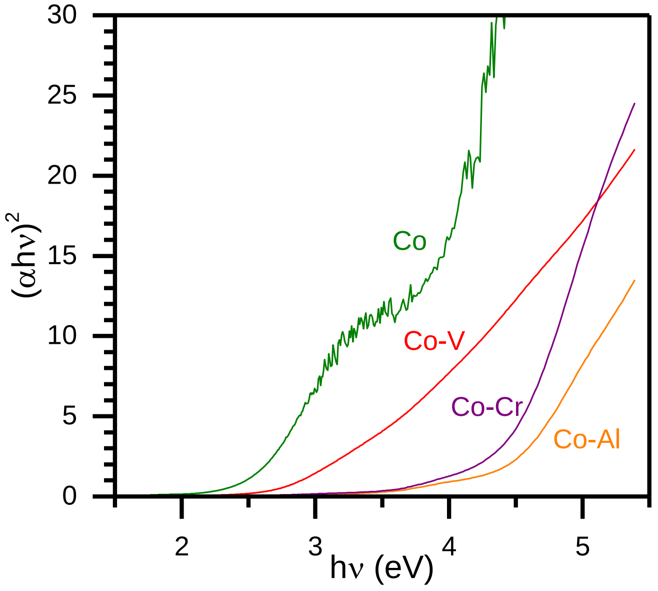

Figure S7 *UV-Vis Data for LHs.* Tauc plot of LH UV-Vis data using r = ½ for direct allowed transitions.

F. Electrochemical Measurement / Electrochemical Quartz Crystal Microbalance (EQCM)

Electrochemical characterization for *ex situ* and *in situ* HE-XRD and XAS studies was performed with a potentiostat (Biologic, SP-300) using a Ag/AgCl reference electrode (BASi) and graphite counter electrode (7 mm dia. Graphite electrode, BioLogic). Electrolyte solutions for all electrochemical studies were purged with Ar for > 15 minutes prior to and throughout electrochemical operation.

For *ex situ* electrochemical characterization, 7 μL aliquots of LH nanoparticle suspensions were dropcast onto glassy carbon electrodes (GCEs, ALS Co. 3mm dia.) and dried under vacuum for > 15 minutes prior to electrochemical measurement. Galvanostatic (constant current) measurements were performed in 0.1 M NaCl (Alfa Aesar, ACS Grade 99.0% min) aqueous electrolyte tuned to a pH of 10 using NaOH. Prior to each galvanostatic measurement, cyclic voltammetry (CV) was performed for all LH samples at a sweep rate of 50 mV/s as presented in Figure S8. CV measurements are consistent with galvanostatic measurements reported in the main text.

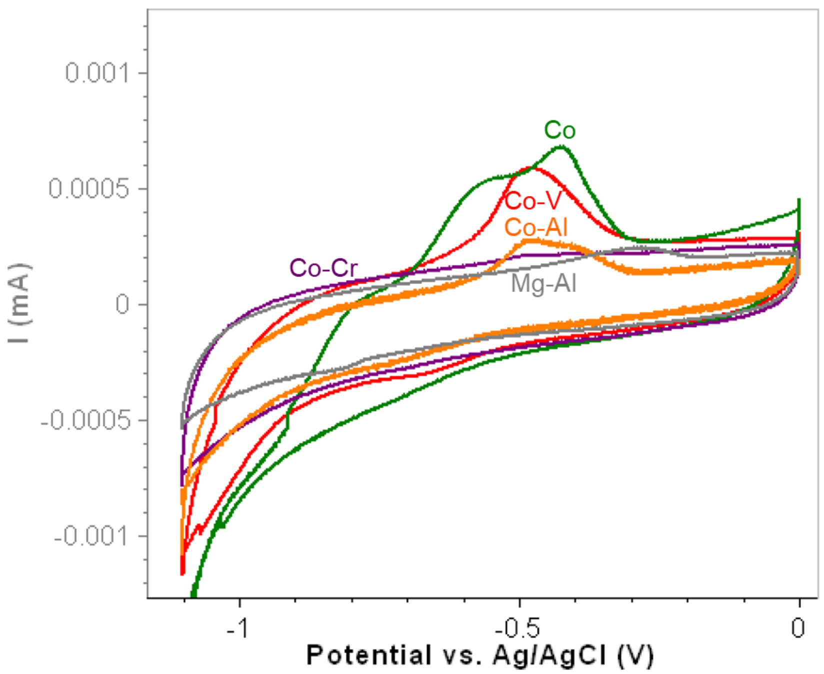

Figure S8 *Cyclic voltammetry measurement of LHs.* Steady state (4th sweep) CV measurements at a sweep rate of 50 mV/s in 0.1M NaCl at a pH of 10 for LHs dropcast on glassy carbon. These measurements were performed immediately prior to the galvanostatic measurements reported in Figure 3 of the main text for each sample.

For EQCM studies, LH nanoparticle suspensions were diluted into equal parts DI water and 20 μL volume aliquots were dropcast onto titanium coated QCM crystals (FilTech, 4.95MHz,14mm), and dried for > 20 minutes under vacuum. LH-coated QCM crystals were loaded into a Q-sense electrochemistry module (Biolin Scientific) using a platinum plate counter electrode and Ag/AgCl reference electrode (Dri-Ref 2SH, World Precision Instruments). 0.1 M NaCl was tuned to a pH of 10 using NaOH and used as the electrolyte solution. The electrolyte was purged for > 15 minutes using Ar prior to use, and kept under continual purge during experimentation. The pH of the source electrolyte was monitored during operation and remained between 10 and 10.5. Electrolyte was used to purge the cell and tubing for > 5 minutes at a flow rate of 0.4 mL/min using a peristaltic pump (Ismatec IPC). Electrolyte flow was stopped during electrochemical characterization to eliminate flow eddies and vibrations present during flow and improve QCM resolution.

During *in situ* HE-XRD a 0.1 M NaBr (ACS Grade, Alfa Aesar) electrolyte was used to enhance the diffraction signal arising from interplanar anions (Br vs. Cl). A continuous electrolyte flow of 0.4 mL/min was maintained during *in situ* HE-XRD electrochemical operation using a peristaltic pump (Ismatec IPC). Fresh electrolyte was used for each experiment, and electrolyte was recirculated during electrochemical characterization. NaBr was also used for XAS studies to be consistent with HE-XRD studies.

G. Inductively Coupled Plasma – Optical Emission Spectroscopy (ICP-OES)

For ICP-OES, LH aqueous suspensions were gravimetrically diluted to 1% concentrations in DI water. We then gravimetrically combined 5 g of 1% dilutions with 5 g of 10% nitric acid. These solutions and DI-water and nitric acid control solutions were submitted to the Laboratory for Environmental and Geological Studies (LEGS) at the University of Colorado Boulder for ICP-OES measurement and analysis.

Synthesis of Mg-Al LHs in this work yielded a bulk M^{2+}:M^{3+} (Mg:Al) molar ratio of 1.7 as measured by inductively coupled plasma optical emission spectroscopy (ICP-OES). This is in agreement with prior work on Mg-Al LDHs indicating stable Mg-Al LDHs with Mg:Al stoichiometric ratios between 1 and 5.[1] Scanning transmission electron microscopy (STEM) with energy-dispersive X-ray spectroscopy (EDS) mapping (STEM-EDS) indicates uniform distribution of Mg and Al in LDH particles as depicted in Figure 3c.

Co-Al and Co-Cr LHs synthesized in this work exhibited M^{2+}:M^{3+} molar ratios of 1.8 and 2.0, respectively, by ICP-OES. These stoichiometric ratios are in agreement with prior work which reports molar ratios of ~2 for these compositions.[27,28] In addition, we also synthesize Co-V LDHs, which to our knowledge have not been reported previously. ICP-OES measurements indicated a bulk Co:V molar ratio of 48:1, corresponding to Co LH ($Co_x(OH)_{2x}Cl$) with ~2% V-doping. However, STEM-EDS analysis of Co-V LHs (See SM Section B) identified particles containing a 3:1 ratio of Co:V, suggesting a mixture of $Co_3V(OH)_8Cl$ and Co LH. A low bulk V concentration is expected considering the favorability of V vacancies in the Co-V structure (See SM Section E).

Despite the low V concentrations in the Co-V LDH, the atomic structure of the Co-V LDH (Figure 1b) is in close agreement with the other LDH structures. We also emphasize that the measured UV-vis band gap of the V-doped cobalt hydroxide structure (2.7 eV) is in close agreement with calculated band gap for a 3:1 Co:V stoichiometric ratio (2.75 eV for Co_3V LDH), and do not agree with calculated band gaps for the $Co(OH)_2$ cobalt hydroxide structure (3.6 eV) indicating that 2% V-doping is sufficient to impact the bulk electronic properties. Although the electronegativity of V (1.63) falls between that of Al and Cr (1.61 and 1.66, respectively),[29] the band gap of the Co-V LDH is dramatically lower than the other structures. We attribute this to differences in the t_{2g}-e_g filling for V vs. Cr.

H. Defect calculations in Co_3V LDH

Presented in Figure S9 are UEB defect plots calculated for the Co_3V LDH structure. These calculations predict that under applied negative bias, Cl^- is stable in the Co_3V LDH structure over a wide potential range, and is not predicted to be removed until a negative bias exceeding -1.6 V vs. Ag/AgCl. The formation of O^{2-} vacancies is predicted to occur before the formation of Cl^- vacancies at a lower negative bias of -1.4 V vs. Ag/AgCl In Co_3V. Additionally, Cl^- insertion (Cl_i) is not predicted to occur until a positive potential exceeding +0.4 V vs. Ag/AgCl. Based on these results, Cl^- will not reversibly incorporate in Co_3V under applied bias. However, the formation of vanadium vacancies, labeled v_V, is predicted to be highly favorable ($\Delta E_f < -2$ eV) over the full range of applied potential, while a reduction of these vacancies is predicted to occur

at negative potentials exceeding -1.4 V. Similarly, cobalt vacancies (v_{Co}) are predicted to be moderately favorable (ΔE_f < -0.2 eV) in Co_3V LDH, with oxidation of these vacancies predicted at positive potentials exceeding -0.1 V vs. Ag/AgCl.

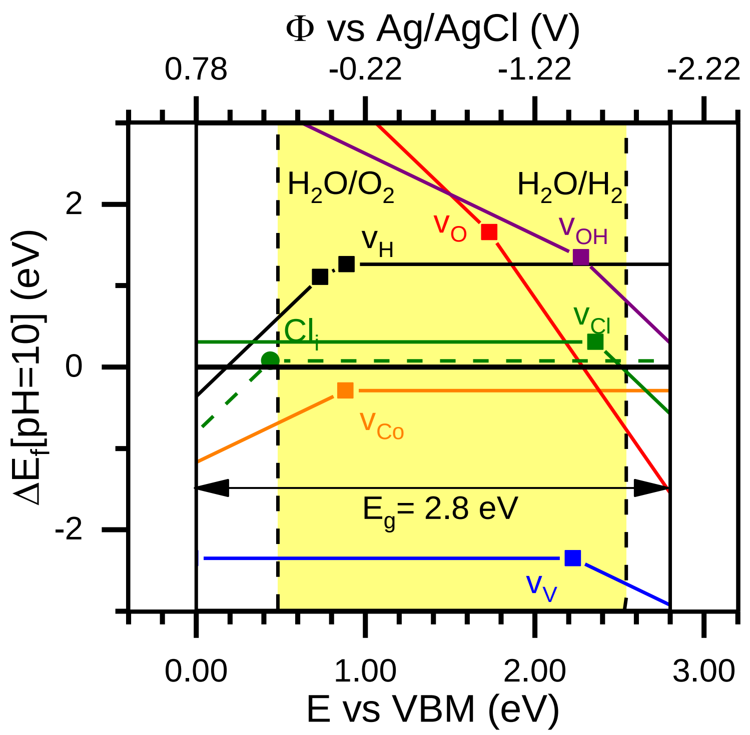

Figure S9 *Defect formation energy versus applied potential for Co_3V LDH.* Calculations on the Co_3V structure suggest that Cl^- insertion will not take place until potentials outside the potential limits for water stability. However, the formation of v_O, v_{Co}, and v_V are all predicted to be favorable within the applied potential window.

I. X-ray Absorption Spectroscopy and Fine Structure Analysis

X-ray absorption spectroscopy (XAS) and subsequent extended X-ray absorption fine structure analysis (EXAFS) was performed using *in situ* XAS measurements during electrochemical characterization at the 10-BM beamline at the APS at Argonne National Lab. 400 µL of LH suspension were dropcast onto nonwoven carbon fiber paper (Fuel Cell Earth, Toray Carbon Paper, ~ 300 µm thickness) in 50 µL aliquots, resulting in a mass loading of ~1 µg. This carbon paper was loaded into a custom polyether ether ketone (PEEK) electrochemical cell with Kapton windows and titanium wire current collector as depicted in other work.[3] A BioLogic SP-300 potentiostat was used for electrochemical control.

EXAFS measurements were performed on the Co K-edge (7.71 keV) in a fluorescence geometry using a 4-element Vortex Silicon Drift Diode. For each measurement a constant voltage was applied to the cell and EXAFS scan were taken from -200 eV to +800 eV relative to the Co K-edge. See Figure S10 for Co K-edge X-ray absorption near-edge spectra (XANES) of the Co-V LDH under various applied biases. Subsequent data processing and EXAFS modeling as performed using the Athena and Artemis programs of the Demeter XAS software package.[30]

EXAFS modeling was first performed on reference Co foil to obtain an S_0^2 value of 0.755 for all subsequent modeling efforts. EXAFS modeling on Co-V LDH at various potentials was performed using theoretical scattering paths from DFT generated structures (see Figure S11 and S12 for the amplitude and real space fitting results, respectively). Given the difficulty in distinguishing Co from V theoretical scattering paths, models were performed by treating all metal scatters as a singular elemental species. EXAFS modeling produced similar Co-O coordination number (~6) and Co-O Debye-Waller factors (~0.008) for each voltage condition, which were then considered as defined constants for subsequent modeling efforts to obtain Co-metal structure and Co-O nearest neighbor distances. All fittings resulted in R-factors of less than 0.02, indicating a high quality fit.

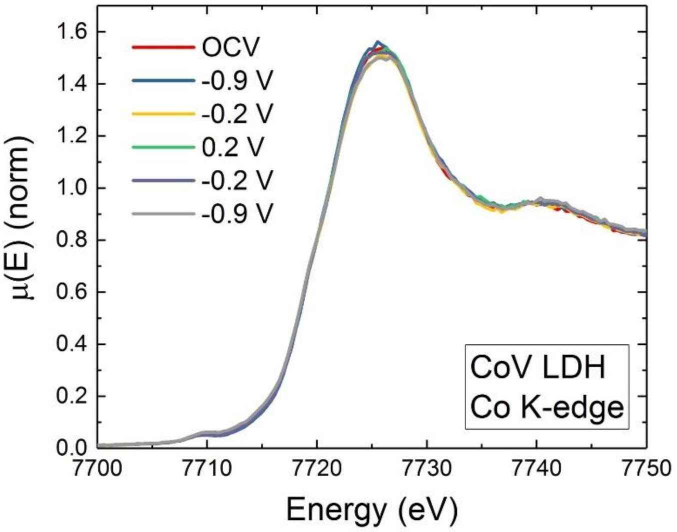

Figure S10 *In-situ electrochemical XANES at the Co K-edge.* XANES measurements at various potentials vs. Ag/AgCl in order of the potential holds applied during each experiment.

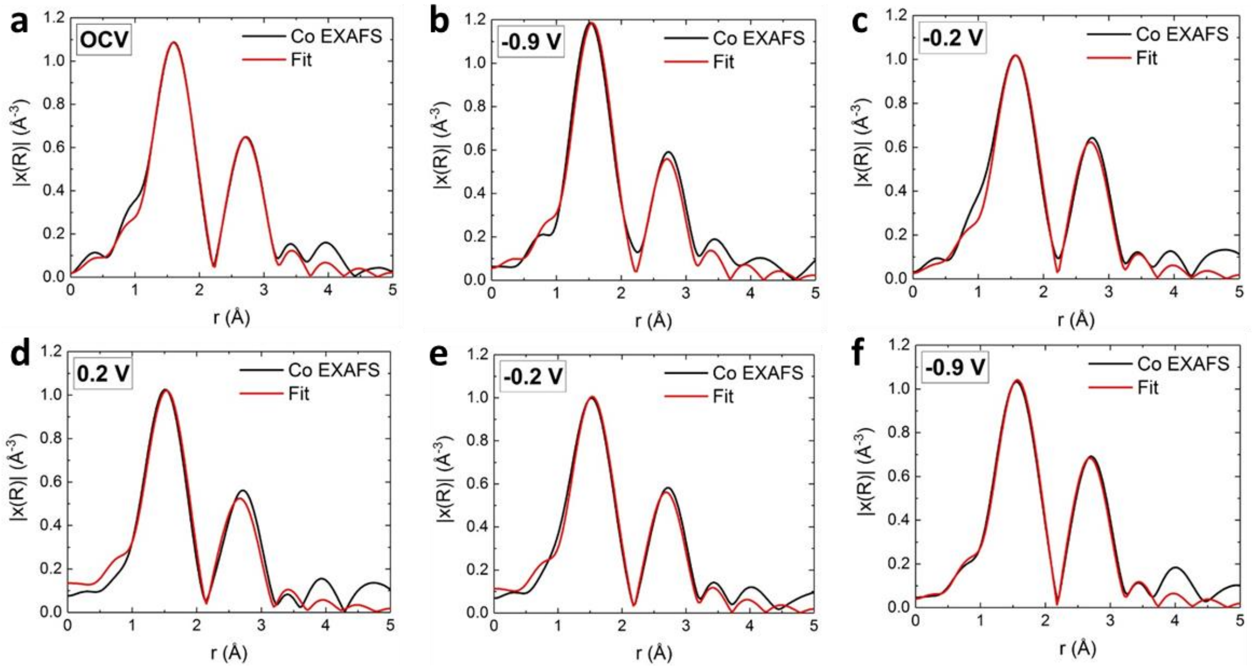

Figure S11 *Co K-edge amplitude EXAFS fitting results.* EXAFS fitting results shown as a function of the amplitude for: a) open circuit voltage; b) -0.9 V; c) -0.2 V, d) 0.2 V, e) -0.2 V and e) -0.9 V. All potentials are referenced against Ag/AgCl and were performed in sequential order.

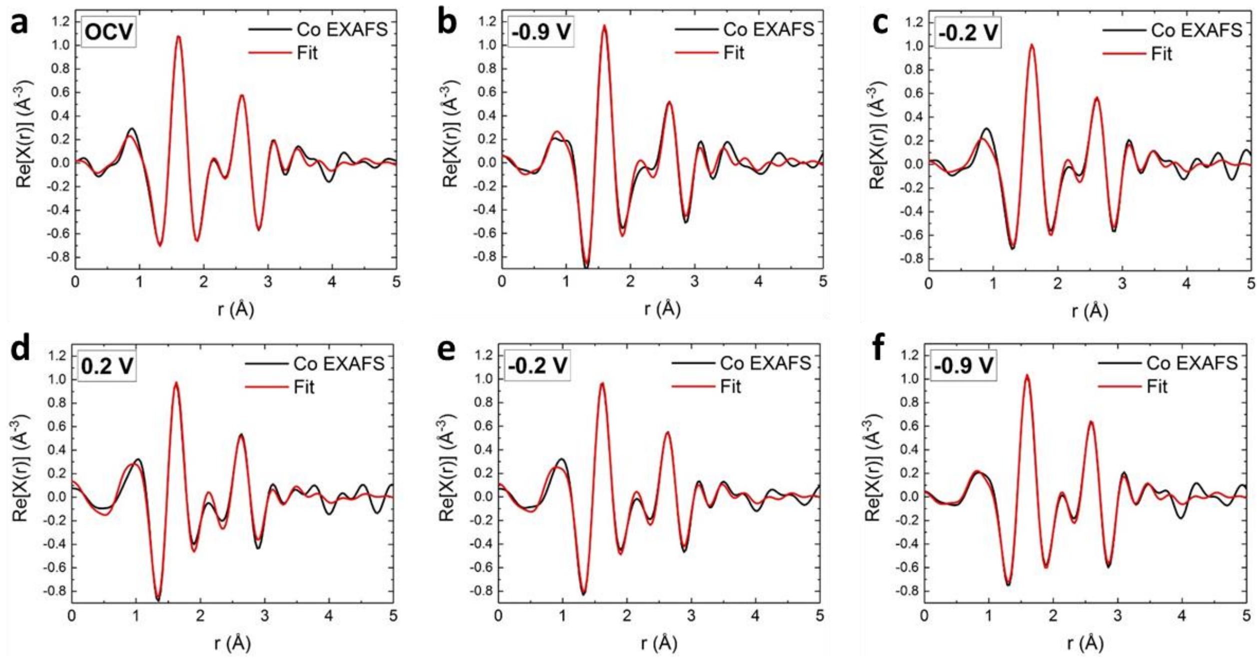

Figure S12 *Co K-edge real-space EXAFS fitting results.* EXAFS fitting results shown in real space for: a) open circuit voltage; b) -0.9 V; c) -0.2 V, d) 0.2 V, e) -0.2 V and e) -0.9 V. All potentials are referenced against Ag/AgCl and were performed in sequential order.

J. References

1. Miyata, S. The Syntheses of Hydrotalcite-Like Compounds and Their Structures and Physico-Chemical Properties I: The Systems Mg^{2+}-Al^{3+}-NO_3^-, -Cl^-, -ClO_4^-, Ni^{2+} and Zn^{2+}. *Clays Clay Miner.* **23,** 369–375 (1975).

2. Xu, Z. P., Stevenson, G., Lu, C.-Q. & Lu, G. Q. M. Dispersion and size control of layered double hydroxide nanoparticles in aqueous solutions. *J Phys Chem B* **110,** 16923–9 (2006).

3. Young, M. J., Bedford, N. M., Jiang, N., Lin, D. & Dai, L. In situ electrochemical high-energy X-ray diffraction using a capillary working electrode cell geometry. *J. Synchrotron Radiat.* **24,** 787–795 (2017).

4. Juhás, P., Davis, T., Farrow, C. L. & Billinge, S. J. L. PDFgetX3: A rapid and highly automatable program for processing powder diffraction data into total scattering pair distribution functions. *J. Appl. Crystallogr.* **46,** 560–566 (2013).

5. Krivovichev, S. V. *et al.* Crystal chemistry of natural layered double hydroxides. 1. Quintinite-2H-3c from the Kovdor alkaline massif, Kola peninsula, Russia. *Mineral. Mag.* **74,** 821–832 (2010).

6. Jain, A. *et al.* Commentary: The Materials Project: A materials genome approach to accelerating materials innovation. *APL Mater.* **1,** 011002 (2013).

7. Izumi, F. Beyond the ability of Rietveld analysis: MEM-based pattern fitting. *Solid State Ionics* **172,** 1–6 (2004).

8. Momma, K. & Izumi, F. VESTA 3 for three-dimensional visualization of crystal, volumetric and morphology data. *J. Appl. Crystallogr.* **44,** 1272–1276 (2011).

9. Aoun, B. Fullrmc, a rigid body reverse monte carlo modeling package enabled with machine learning and artificial intelligence. *J. Comput. Chem.* **37,** 1102–1111 (2016).

10. Young, M. J., Holder, A. M., George, S. M. & Musgrave, C. B. Charge Storage in Cation Incorporated α-MnO_2. *Chem. Mater.* **27,** 1172–1180 (2015).

11. Young, M. J., Schnabel, H.-D., Holder, A. M., George, S. M. & Musgrave, C. B. Band Diagram and Rate Analysis of Thin Film Spinel $LiMn_2O_4$ Formed by Electrochemical Conversion of ALD-Grown MnO. *Adv. Funct. Mater.* **26,** 7895–7907 (2016).

12. Young, M. J., Holder, A. M. & Musgrave, C. B. The Unified Electrochemical Band Diagram Framework: Understanding the Driving Forces of Materials Electrochemistry. *Adv. Funct. Mater.* **1803439,** 1803439 (2018).

13. Blöchl, P. E. Projector augmented-wave method. *Phys. Rev. B* **50,** 17953 (1994).

14. Kresse, G. & Furthmüller, J. Efficient iterative schemes for ab initio total-energy calculations using a plane-wave basis set. *Phys. Rev. B. Condens. Matter* **54,** 11169–11186 (1996).

15. Kresse, G. From ultrasoft pseudopotentials to the projector augmented-wave method. *Phys. Rev. B* **59,** 1758–1775 (1999).

16. Perdew, J., Burke, K. & Ernzerhof, M. Generalized Gradient Approximation Made Simple. *Phys. Rev. Lett.* **77,** 3865–3868 (1996).

17. Schimka, L., Harl, J. & Kresse, G. Improved hybrid functional for solids: The HSEsol functional. *J. Chem. Phys.* **134,** 024116 (2011).

18. Alkauskas, A. & Pasquarello, A. Band-edge problem in the theoretical determination of defect energy levels: The O vacancy in ZnO as a benchmark case. *Phys. Rev. B* **84,** 1–11 (2011).

19. Pacchioni, G. Modeling doped and defective oxides in catalysis with density functional theory methods: room for improvements. *J. Chem. Phys.* **128,** 182505 (2008).

20. Sherman, D. M. Electronic structures of iron(III) and manganese(IV) (hydr)oxide minerals: Thermodynamics of photochemical reductive dissolution in aquatic environments. *Geochim. Cosmochim. Acta* **69,** 3249–3255 (2005).

21. Schott, H. Relationship between zero point of charge and solubility product for hydroxides of polyvalent cations. *J. Pharm. Sci.* **66,** 1548–1550 (1977).

22. Sideris, P. J., Nielsen, U. G., Gan, Z. & Grey, C. P. Mg/Al Ordering in Layered Double Hydroxides Revealed by Multinuclear NMR Spectroscopy. *Science* **321,** 113–117 (2008).

23. Fall, C., Binggeli, N. & Baldereschi, A. Deriving accurate work functions from thin-slab calculations. *J. Phys. Condens. Matter* **11,** 2689–2696 (1999).

24. Zhang, F. *et al.* Crystal-face-selective supporting of gold nanoparticles on layered double hydroxide as efficient catalyst for epoxidation of styrene. *ACS Catal.* **1,** 232–237 (2011).

25. Perdew, J. *et al.* Restoring the Density-Gradient Expansion for Exchange in Solids and Surfaces. *Phys. Rev. Lett.* **100,** 136406-1-136406–4 (2008).

26. Stevanović, V., Lany, S., Ginley, D. S., Tumas, W. & Zunger, A. Assessing capability of semiconductors to split water using ionization potentials and electron affinities only. *Phys. Chem. Chem. Phys.* **16,** 3706–14 (2014).

27. Dong, C. *et al.* Rational design of cobalt–chromium layered double hydroxide as a highly efficient electrocatalyst for water oxidation. *J. Mater. Chem. A* **4,** 11292–11298 (2016).

28. Carrado, K. A., Kostapapas, A. & Suib, S. L. Layered double hydroxides (LDHs). *Solid State Ionics* **26,** 77–86 (1988).

29. *CRC Handbook of Chemistry and Physics (Internet Version 2017)*. (CRC Press/Taylor & Francis, 2017).

30. Ravel, B. & Newville, M. ATHENA, ARTEMIS, HEPHAESTUS: Data analysis for X-ray absorption spectroscopy using IFEFFIT. *J. Synchrotron Radiat.* **12,** 537–541 (2005).
